# A mathematical and computational model of the calcium dynamics in *Caenorhabditis elegans* ASH sensory neuron

**DOI:** 10.1371/journal.pone.0201302

**Published:** 2018-07-26

**Authors:** Ehsan Mirzakhalili, Bogdan I. Epureanu, Eleni Gourgou

**Affiliations:** 1 Mechanical Engineering Department, University of Michigan, Ann Arbor, Michigan, United States of America; 2 Department of Internal Medicine, Division of Geriatrics, Medical School, University of Michigan, Ann Arbor, Michigan, United States of America; Cinvestav-IPN, MEXICO

## Abstract

We propose a mathematical and computational model that captures the stimulus-generated Ca^2+^ transients in the *C*. *elegans* ASH sensory neuron. The rationale is to develop a tool that will enable a cross-talk between modeling and experiments, using modeling results to guide targeted experimental efforts. The model is built based on biophysical events and molecular cascades known to unfold as part of neurons' Ca^2+^ homeostasis mechanism, as well as on Ca^2+^ signaling events. The state of ion channels is described by their probability of being activated or inactivated, and the remaining molecular states are based on biochemically defined kinetic equations or known biochemical motifs. We estimate the parameters of the model using experimental data of hyperosmotic stimulus-evoked Ca^2+^ transients detected with a FRET sensor in young and aged worms, unstressed and exposed to oxidative stress. We use a hybrid optimization method composed of a multi-objective genetic algorithm and nonlinear least-squares to estimate the model parameters. We first obtain the model parameters for young unstressed worms. Next, we use these values of the parameters as a starting point to identify the model parameters for stressed and aged worms. We show that the model, in combination with experimental data, corroborates literature results. In addition, we demonstrate that our model can be used to predict ASH response to complex combinations of stimulation pulses. The proposed model includes for the first time the ASH Ca^2+^ dynamics observed during both "on" and "off" responses. This mathematical and computational effort is the first to propose a dynamic model of the Ca^2+^ transients' mechanism in *C*. *elegans* neurons, based on biochemical pathways of the cell's Ca^2+^ homeostasis machinery. We believe that the proposed model can be used to further elucidate the Ca^2+^ dynamics of a key *C*. *elegans* neuron, to guide future experiments on *C*. *elegans* neurobiology, and to pave the way for the development of more mathematical models for neuronal Ca^2+^ dynamics.

## Introduction

The use of Ca^2+^ transients to indirectly assess a neuron's activation is a well-established approach [1–3] despite its limitations and the caution needed when drawing conclusions about the neuron's concurrent depolarization [4–8]. *C*. *elegans* in particular has been proved ideal for applying imaging techniques to monitor stimulus-evoked Ca^2+^ transients in a variety of neurons [2,9–14], in freely moving [10,15] as well as in immobilized worms, by using either traditional approaches [3,4,16,17] or advanced methods [2,18–22]. The ASH polymodal neuron is the subject of numerous such studies [18,19,22,23], due to its key importance as a nociceptor for the worms' survival and also because it is the starting point for a plethora of downstream neuronal events. The use of microfluidic chips for worm immobilization and stimulus delivery has revealed a second peak in the ASH Ca^2+^ transients, occurring upon withdrawal of the stimulus (the "off" response) [19,24,25], in addition to the first peak, which occurs upon delivery of the stimulus (the “on” response).

The “on” and “off” responses that ensue in the ASH neuron upon its stimulation are the object of several studies, which explore the connection between Ca^2+^ transients, neuronal behavior [6,24–26] and synaptic output of the ASH neuron to downstream neurons [11,19,27]. In particular, the connection between Ca^2+^ transients in the ASH neuron and *C*. *elegans* behavior has been the object of several studies that suggest an interesting correlation not only between the "on" response and specific behaviors [28–30], but also between the "off" response and avoidance behavior [24]. This leads to the conclusion that all features of the Ca^2+^ dynamics in the ASH neuron participate in fine-tuning the worm's rich behavioral repertoire.

Ca^2+^ transients [3] have been studied in ASH neurons in the context of different biological or environmental conditions, including aging [18,23], oxidative stress [12,22], food availability [30] and oxygen concentration [31]. Extended efforts have been made to decipher the molecular players involved [3,4,24,26,32]. At the same time, mathematical modeling of Ca^2+^ dynamics has been performed in a variety of organisms and cells, but the literature is sparse and far from complete for mathematical modeling of Ca^2+^ response in *C*. *elegans* neurons [28,33,34]. Work conducted by Kato and colleagues [25] has focused on the temporal responses of ASH and AWC to flickering stimuli, developing a phenomenological model that explains selected features of Ca^2+^ dynamics using ordinary differential equations. However, that work does not include the ASH "off" response, and the model does not account for the dynamics of the molecular players involved.

We propose a mathematical and computational model that is based on biochemical pathways and for the first time encompasses the Ca^2+^ dynamics observed during both the "on" and "off" responses in the *C*. *elegans* ASH neuron. The approach integrates biophysical models that describe several aspects of the overall Ca^2+^ signaling mechanism and it merges them into an inclusive model forged by novel phenomenological adjustments and known biochemical motifs. Thus, the model succeeds in capturing the Ca^2+^ dynamics in ASH neuron with excellent fidelity. Moreover, we show how the model can be used to suggest potential changes in molecular components that can explain modifications in Ca^2+^ dynamics due to aging and oxidative stress to guide future experiments. Lastly, we demonstrate how the proposed model can be used to predict Ca^2+^ transients in ASH neuron when delivering arrays of complex stimuli.

## Results

**Fig 1:**
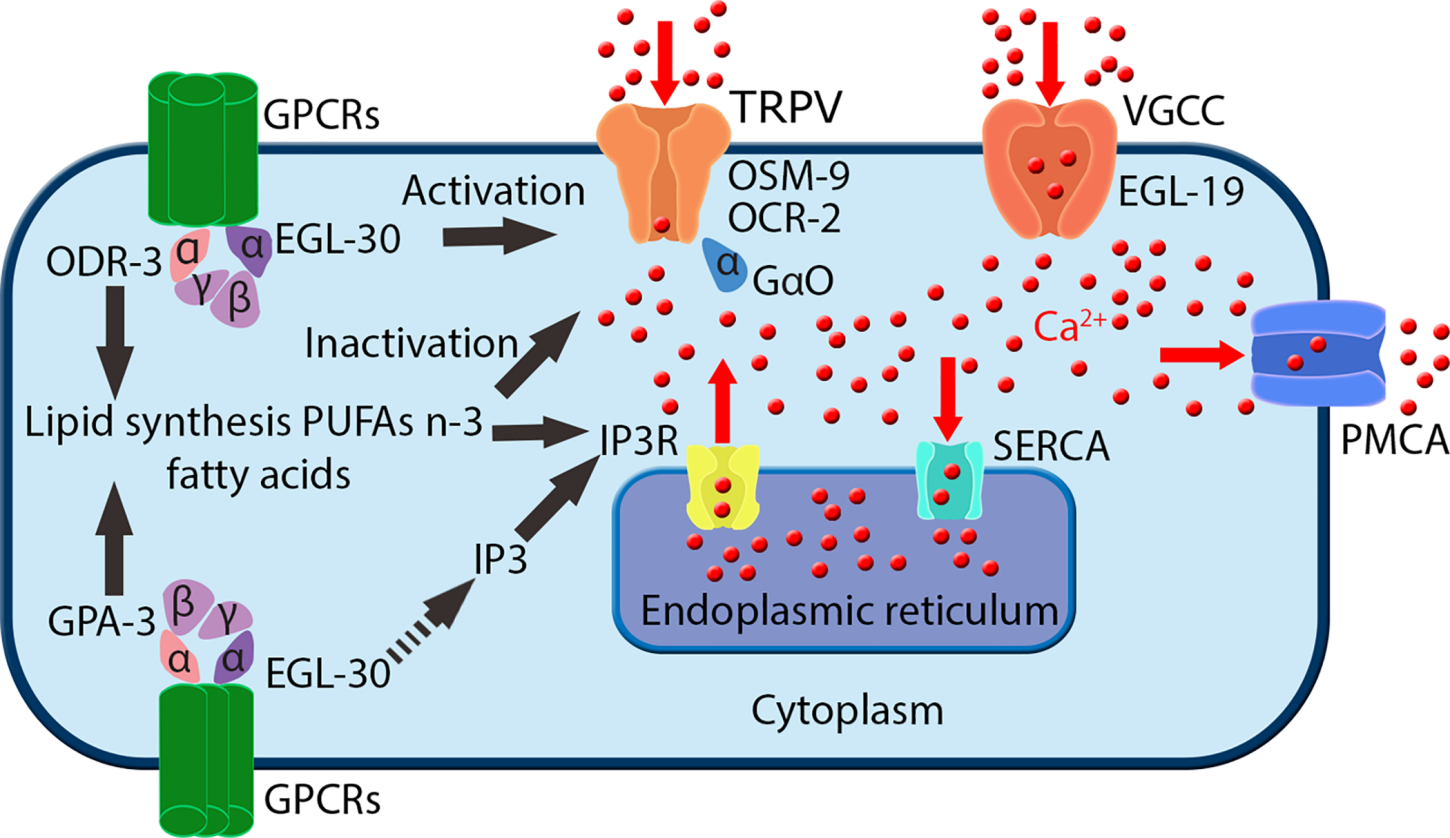
Molecular components of the Ca^2+^ homeostatic machinery that are included in the proposed mathematical model. GPCRs: G-protein coupled receptors, as the ones that are activated in ASH neuron by hyperosmotic stimuli; ODR-3, EGL-30, GPA-3: G-proteins coupled with the receptors, participating in signal transduction to downstream ion channels; OSM-9, OCR-2: molecular elements of the TRPV channels, the main cation channels through which Ca^2+^ flows into the neuron upon its stimulation; GαO: G-protein coupled with TRPVs; EGL-19: molecular component of the VGCCs, the L-type voltage gated Ca^2+^ channels, activated by the changed membrane potential due to ion influx upon neuronal activation; PMCA: plasma membrane Ca^2+^ ATPase, the main pump responsible for transporting Ca^2+^ into the extracellular space; SERCA: sarco-endoplasmic reticulum Ca^2+^ ATPase, which transports Ca^2+^ into the intracellular stores; IP3: 3-phopsho-inositol, secondary messenger participating in Ca^2+^ signaling events; IP_3_R: IP_3_ receptors, glycoprotein complex acting as a Ca^2+^ channel activated by IP_3_, abundant on the endoplasmic reticulum (ER) membranes. GPCRs, ODR-3, EGL-30, GPA-3, OSM-9, OCR-2: not modeled individually; model parameters that account for these molecular components are P_0_, P_1_ and P_2_, see Methods. Lipid synthesis, PUFAs, fatty acids, TRPV activation, TRPV inactivation events: modeled as O (activated) and I (inactivated) probabilities, see Methods. Included in the model and not depicted here: J_LEAK_ and J_LEAK, ER_, which represent the constant influx of Ca^2+^ into the cytoplasm from extracellular space and ER, respectively, through other mechanisms, see Methods.

The pathways and molecules included in the proposed model are portrayed in Fig 1. A detailed description of their contribution in the generation of Ca^2+^ transients and their connection with the rest of the model components is given in the Discussion.

### Accuracy and effects of knocking out components of the model

**Fig 2:**
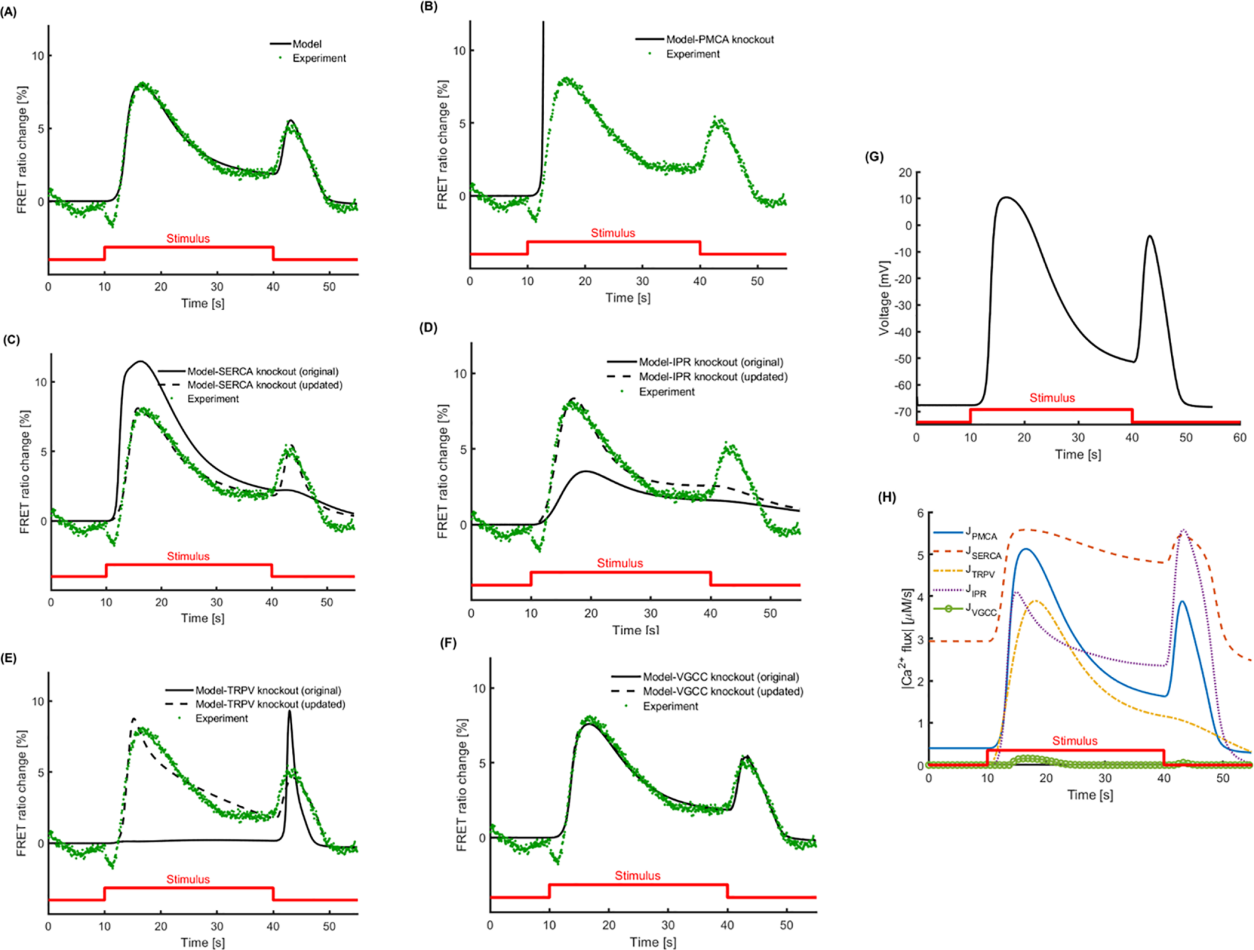
The proposed mathematical model can capture the Ca^2+^ dynamics observed in young (Day 1) unstressed worms, when ASH sensory neuron is stimulated by hyperosmotic solution (glycerol 1M). (A) The model matches the Ca^2+^ transients, as recorded experimentally, including all key features (time and magnitude of peaks, rising and decay slopes) for the "on" response (upon delivery of the stimulus) and the "off" response (upon withdrawal of the stimulus). (B-F) Different components of the model are knocked out (in silico knock-out) to investigate their impact on the model-generated results. For the knockout results with original parameters (solid line), all the parameters are kept the same as in (A), except for the knockout component that is removed from the mathematical model. For the knockout results with updated parameters (dashed line), the hybrid optimization algorithm is run again trying to find an updated set of parameters which can explain the experimental Ca^2+^ dynamics, after the knocked-out component has been removed from the mathematical model. (B) When PMCA is removed, Ca^2+^ is not pumped out of the cell and the mathematical model fails. Changes in the parameters in any of the model components cannot compensate for PMCA knockout in the updated model. (C) When SERCA is removed, the original model fails to show the "off" response and features of the "on" response are also affected. Updating the parameters restores most of Ca^2+^ transients' features, except for the decaying slopes. (D) Removal of IPR affects all the features of "on" response and the "off" response completely vanishes. Updating the parameters restores the "on" response partly but fails to rescue the "off" response. (E) TRPV knockout for the original model does not show any "on" response, while "off" response is amplified. When the parameters are updated, both "on" and "off" responses are partially restored. (F) VGCC *in silico* knock-out does not affect significantly the results of the model, original or updated. The dashed and continuous lines coincide; the dashed line is distinguishable around the peak of the “on” response. (G) The phenomenological model used to describe the voltage response shows a graded response when the stimulus is applied. A weaker graded voltage response is also observed when the stimulus is removed. (H) The Ca^2+^ fluxes for different components in the original model show how much each component contributes to the overall dynamics. *J*_*PMCA*_ is the flux of Ca^2+^ by PMCA, plasma membrane Ca^2+^ ATPase, the main pump responsible for transporting Ca^2+^ into the extracellular space; *J*_*SERCA*_ is the flux of Ca^2+^ by SERCA, sarco-endoplasmic reticulum Ca^2+^ ATPase, which transports Ca^2+^ into the intracellular stores; *J*_*TRPV*_ is the flux of Ca^2+^ by TRPV, transient receptor potential-vallinoid channels that are responsible for initial influx of Ca^2+^ into the cell; *J*_*IPR*_ is the flux of Ca^2+^ IP_3_ receptors from the stores, which are the main contributor to the “off” response; and *J*_*VGCC*_ is the flux of Ca^2+^ through voltage gated Ca^2+^ channels.

As a first step, we explore whether our model can capture the dynamics of the Ca^2+^ transients which occur when the ASH neuron of young unstressed worms is stimulated by a hyperosmotic solution (Fig 2A). Our results show that the model matches well the special and critical features of the Ca^2+^ transients, including magnitude and time of peaks, rising and decaying slopes for both “on” and “off” responses. The representation of the stimulus as a square pulse corresponds to the way the hyperosmotic solution is delivered to the worm’s nose in the microfluidic device [2,18,19,35].

One way to explore if a component of the model is necessary is to knock out that component and look for parameter sets that can still capture the complete response of the cell. To this end, we removed different components of the model, one at a time, to investigate their contribution to the predicted Ca^2+^ dynamics (Fig 2B–2F). For each of these *in silico* knockouts, two cases were considered: i) the original case, in which after the knockout component is removed the values for all parameters remain the same as in the model that generates the output shown in Fig 2A, and ii) the updated case, in which after the knockout component is removed, we apply anew the hybrid optimization algorithm to estimate again the model parameters.

We use the case of PMCA, which leads to expected results, as the first knock out, to illustrate the purposefulness of the *in silico* knock out approach. We find that the model completely diverges without the PMCA (Fig 2B), since all the Ca^2+^ that flows in upon delivery of the stimulus, remains in the neuron. Moreover, since the PMCA pump is the only component in the model that actively removes excessive Ca^2+^ out of the neuron, changing the value of any other parameter cannot compensate for the PMCA knockout; therefore, no updated solution can be reached when the PMCA is knocked out.

Next, when the SERCA pump is removed (Fig 2C), Ca^2+^ that enters the cytoplasm is not pumped into the ER. The original model with a knocked out SERCA accounts for more Ca^2+^ in the cytoplasm, compared to the results shown in Fig 2A, while at the same time the "off" response is absent. In the updated model, where the parameters of other components are adjusted, the overall results are improved. However, the final return of cytoplasmic Ca^2+^ to its initial levels is still not completely restored, and the rising slope of the "off" response is captured with a slight time lag.

Removing IPRs from the model (Fig 2D) completely alters the features of the Ca^2+^ transients. The magnitude of the “on” response decreases dramatically, and the "off" response vanishes. The updated parameters can recover features of the “on” response, but they fail to yield the “off” response.

The “on” response completely disappears when TRPV channels are knocked out (Fig 2E). Interestingly, the “off” response remains present and strong in the absence of TRPV channels. In the updated model, the adjusted parameters yield the “on” response, even without TRPV channels. However, the updated model without the TRPV channels accounts poorly for the magnitude of the "on" response and, most importantly, it fails to capture the dynamics of its decaying slope.

Elimination of VGCCs from the model (Fig 2F) does not affect its predictions substantially. This is reflected equally in the outputs of the original and the updated version of the model, as shown in Fig 2G.

We also explored the Ca^2+^ fluxes from each model component (Fig 2H). The fluxes associated with PMCA and SERCA are not zero before applying the stimulus, because they need to keep Ca^2+^ flux in balance, despite the leaks across the cell membrane and ER membrane. TRPV channels respond quickly when the stimulus is presented, which leads to higher activity of pumps to balance the excessive Ca^2+^. IPRs also follow TRPVs but their main contribution is to the “off” response when the stimulus is removed. VGCCs respond to the stimulus with a significant lag compared to the other components of the model, because they require a large amount of Ca^2+^ to enter the cell to induce their activation. Hence, VGCCs act like a secondary mechanism, with a weak contribution to total Ca^2+^ influx. Moreover, it is shown that TRPVs do not contribute to the “off” response at all (see also Fig 2E). In addition, it takes time for most of the fluxes to return to or approach their initial equilibrium state after the stimulus is removed. Therefore, a different dynamical response is expected if the first stimulus is followed by a second pulse after a short interval. We explore such scenarios as well.

### The model captures Ca^2+^ dynamics in aged and stressed worms

**Fig 3:**
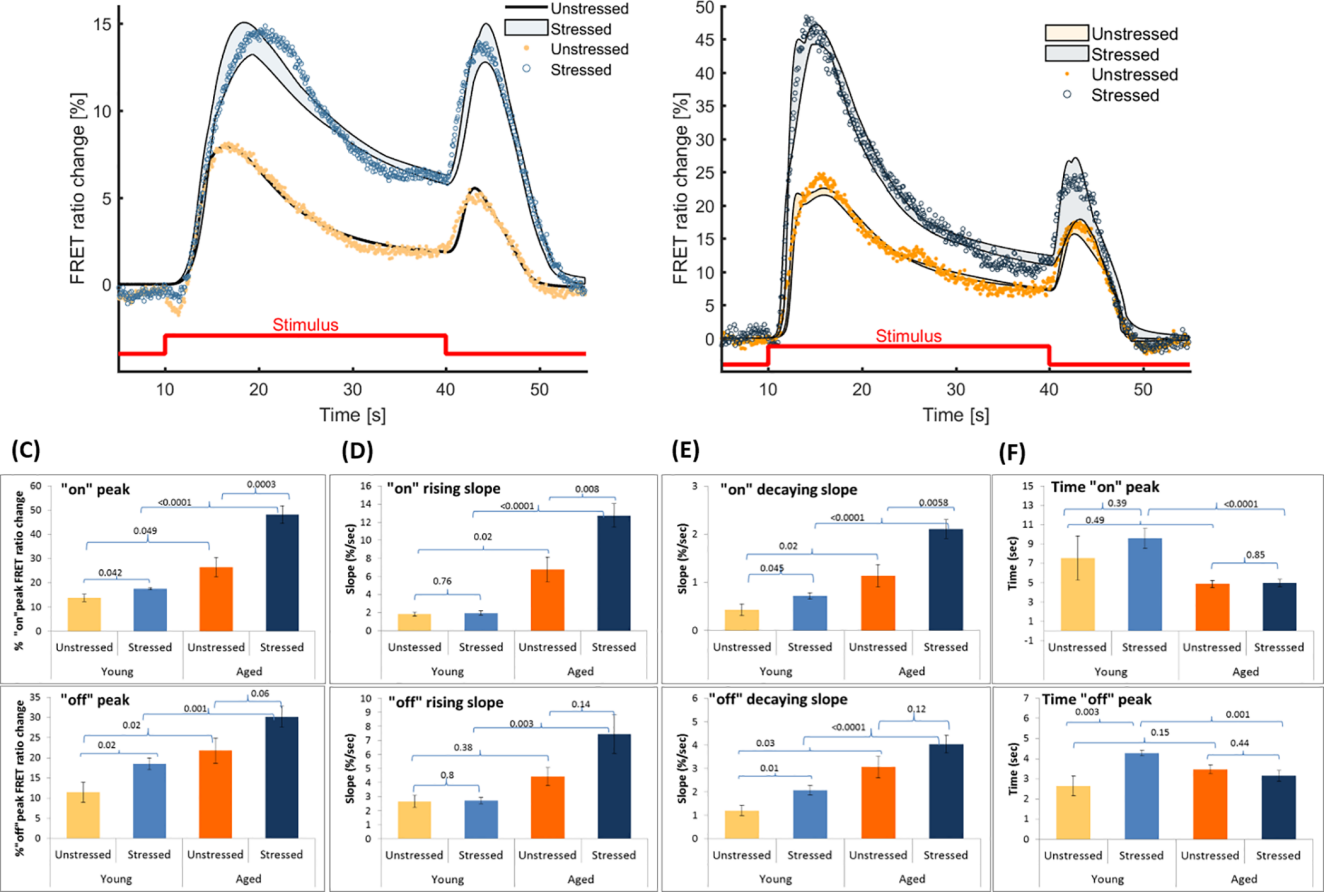
The proposed mathematical model can capture the stimulus-induced changes in the Ca^2+^ dynamics in the case of aged (Day 5) or previously exposed to oxidative stress (stressed) animals. (A) The parameter set for young (Day 1) unstressed worms is used as a reference point to detect changes in the parameters that can explain the stimulus-evoked Ca^2+^ dynamics in treated worms of the same age. The model results shown correspond to all plausible solutions. (B) Similar to (A), the parameter set for young (Day 1) unstressed worms is used to detect changes in the parameters that can explain the stimulus-evoked Ca^2+^ dynamics in aged (Day 5) unstressed and stressed worms. The modeling results correspond to all plausible solutions. Red line represents the stimulus pulse delivered (duration: 30sec). Experimental data originally presented in Gourgou and Chronis, 2016. (C-F) Key features of the Ca^2+^ transients, as recorded experimentally and presented in (A) and (B); (C) the peak of the "on" (top) and "off" (bottom) response, (D) the rising slope of the "on" (top) and the "off" (bottom) response, (E) the decaying slope of the "on" (top) and the "off" (bottom) response, (F) the time needed to reach the peak of "on" (top) and "off" (bottom) response. Error bars indicate standard error of mean, p-values of Student t-test shown in chart area. Representative results of 17–35 individual experiments. (C) top panel and (D) top panel are modified from Gourgou and Chronis, 2016.

The parameters values in the parameter set found to capture the Ca^2+^ dynamics in young unstressed worms (Fig 2A) are used as reference case. Using them as a starting point we can obtain different plausible parameter values that explain Ca^2+^ transients for young stressed (Fig 3A), aged unstressed and aged stressed worms (Fig 3B).

We use the parameters for young unstressed worms to initiate the search for plausible solutions in the other three cases so that we can explore the impact of oxidative stress and age on the model parameters. The experimental data for young stressed worms lie well within the bounds of the results predicted by the mathematical model, except for the time right after the “on” response, as well as some time during the plateau (Fig 3A). In these two short periods the model seems not to follow exactly the stabilization in Ca^2+^ concentration. In the case of aged worms, experimental data falls within the bounds of the results predicted by the mathematical model, with plausible parameter sets for both unstressed and stressed worms (Fig 3B). Most of the model generated variations for aged worms occur around the “off” response, for both unstressed and stressed animals.

In parallel, we quantify key features of the experimentally recorded Ca^2+^ transients (Fig 3C–3F, top and bottom panels) to identify how they change due to oxidative stress and aging, and eventually correlate them with the model parameters. Results show that oxidative stress alone, as revealed when young worms are tested, affects the peak of both the “on” (Fig 3C, top) and “off” (Fig 3C, bottom) responses, as well as their decaying slopes (Fig 3E, top, bottom). Oxidative stress results also in faster occurrence of the “off” response (Fig 3F, bottom). Aging affects the peak of “on” and “off” responses (Fig 3C, top, bottom) and the rising slope of the “on” response (Fig 3D, top) as well as the decaying slopes of both responses (Fig 3E, top, bottom). The combination of aging and stress has an impact on the rising slopes of both responses (Fig 3D, top, bottom) and on how fast they occur (Fig 3F, top, bottom).

### Parameters that contribute to the modified Ca^2+^ dynamics: Model sensitivity

**Fig 4:**
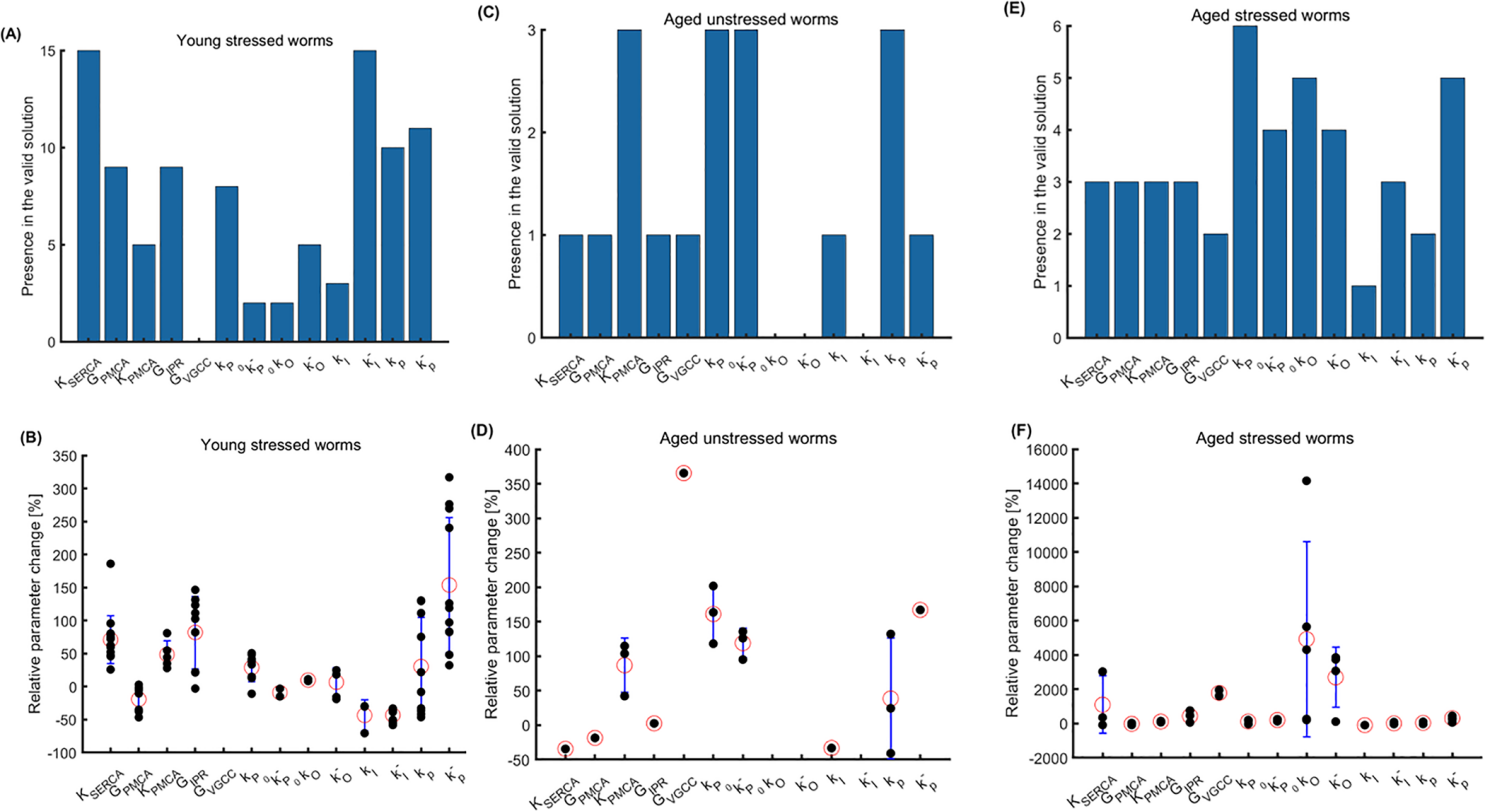
The effects of aging and oxidative stress treatment on stimulus-evoked Ca^2+^ transients can be explained by changing values of the parameter set for young unstressed worms (reference case). The frequency by which each of the selected parameters appears in all plausible combinations of solutions is shown in (A) for young stressed worms (15 plausible combinations), in (C) for aged unstressed worms (3 plausible combinations), and in (E) for aged stressed worms (6 plausible combinations). The dot plots in (B), (D), and (F) show the relative changes in the parameters compared to the respective parameters for young untreated worms. Each dot corresponds to a plausible solution; red circles indicate the mean; error bars represent standard deviation.

Each plausible parameter set obtained from the multitude of possible initial populations in the hybrid optimization algorithm contains combinations of parameters that are different from the reference case (young unstressed worms). The times each parameter is present in a plausible parameter set for young stressed (total of 15 plausible sets), aged unstressed (total of 3 plausible sets) and stressed (total of 6 plausible sets) worms is shown in Fig 4A, 4C and 4E. The absence of a bar for a parameter (for example, VGCC is not present in Fig 4A) does not indicate that the specific parameter remined unchanged at the condition studied (i.e., age and/or oxidative stress). Rather, it means that none of the different combinations that constitute plausible solutions for the specific case (e.g., regarding Fig 4A, stress in young worms), contains this specific parameter. As shown in the dot plots in Fig 4B, 4D and 4F, the values of each parameter in all plausible sets in which it is included may vary substantially (e.g., *k*_*O*_, Fig 4F) or not (e.g., *k*_*p*_, Fig 4B). Notably, in the case of aged stressed worms (Fig 4F) the parameters included in the plausible solutions that are changed compared to young unstressed worms, are increased hundreds of times (e.g., 4000% for $k_{o}^{-}$, 6000% or even 14000% for *k*_*o*_ Fig 4F). In the case of young stressed and aged unstressed worms, the altered parameters increase only by up to ~350% (Fig 4B and 4D), and sometimes they even decrease, compared to young unstressed animals (e.g. G_SERCA_ and k_o_^-^ Fig 4B, K_SERCA_ and k_I_ in Fig 4D).

**Fig 5:**
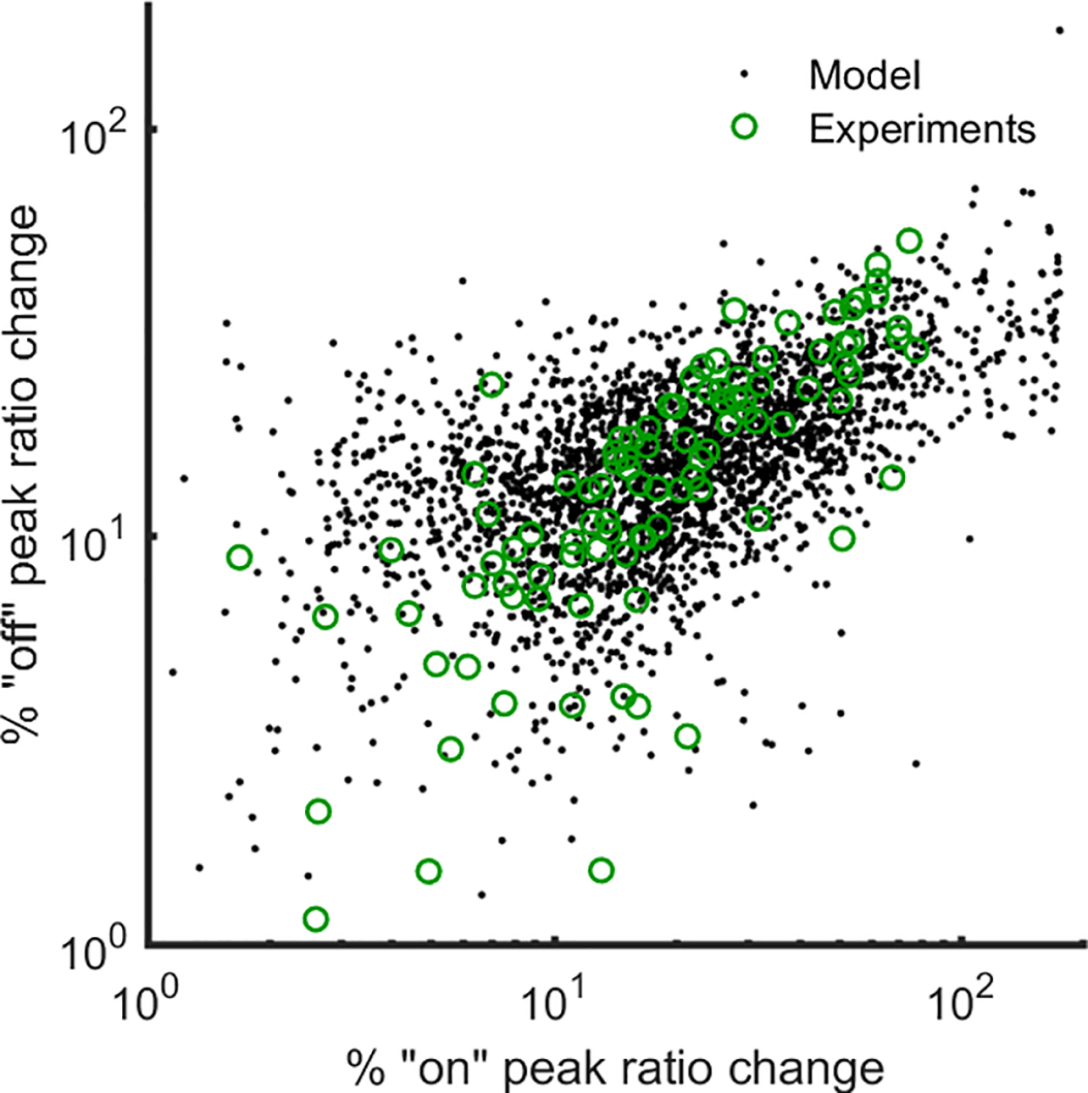
Sensitivity of transients on parameter models. Open green circles represent all the experimental results, including young unstressed, young stressed, aged unstressed, and aged stressed worms. For each plausible parameter set 100 samples are created, in which the parameter values are randomly perturbed by ±25%. For each sample, the maximum magnitudes of the “on” and “off” responses are recorded and plotted as filled black circles. The distribution of randomly perturbed model results shows that the mathematical model can capture the variations that are observed in experimental results. Moreover, the modeling results are observed to be dense/sparse where the experimental results are dense/sparse.

The overall sensitivity of the model parameters can be visualized by a sensitivity plot (Fig 5). For each of the plausible parameter sets, the parameter values are randomly perturbed by ±25%, and the peaks of the “on” and “off” responses are plotted. Results suggest that the model can predict the different behaviors observed in experiments, as shown by the approximate overlap between the model-generated and the experimental results. Moreover, the density of the model-generated data is high where the experimental data points are dense and is low where the experimental data points are sparse. This illustrates that the model generates more results in the regions where most experimental results are recorded.

### Using the model to predict Ca^2+^ dynamics in the case of complex stimuli

**Fig 6:**
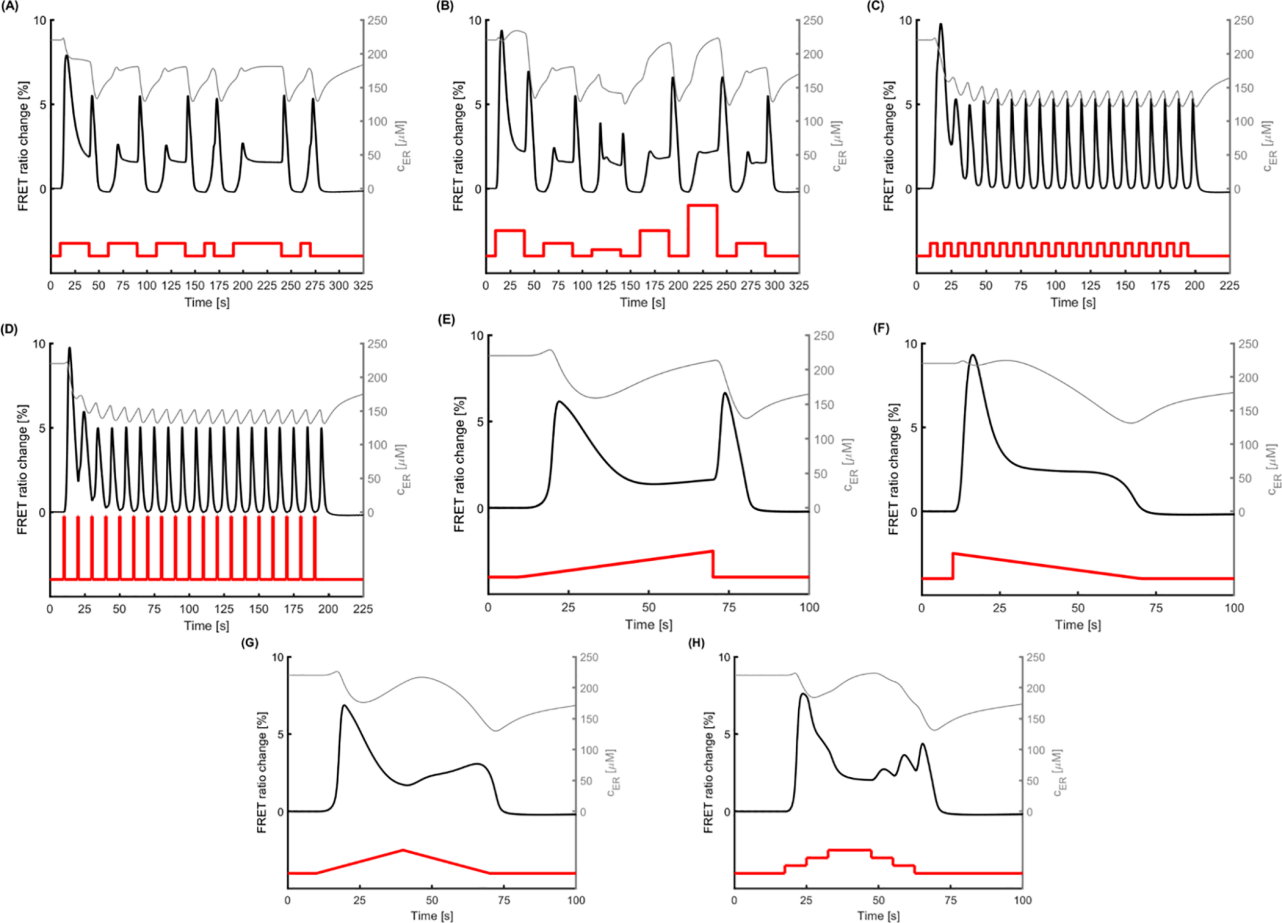
The proposed mathematical model can be used to investigate *in silico* the Ca^2+^ transients which would occur due to complex stimuli, challenging to implement experimentally. The parameters for young (Day 1) unstressed worms (reference case) are used to generate all the results shown in this figure. The left y-axis shows % FRET ratio change and the right y-axis shows the Ca^2+^ concentration in ER. (A) A sequence of stimuli with the same magnitude and different durations is applied. Consecutive stimuli lead to weaker "on" response while the "off" response is less affected. (B) A sequence of stimuli with same duration and different magnitudes is applied. Stronger stimulus leads to larger "off" response for successive stimuli while the "on" response does not increase when the stimulus strength increases. (C) A flickering stimulus results in a Ca^2+^ transient in which an array of single consecutive peaks is observed. (D) A flickering stimulus with the same frequency as (C). The pulses are stronger than the ones in (C), but they are shorter so that the area underneath each plus is the same for each pulse in C and D. The Ca^2+^ transients in (C) and (D) are almost identical. (E) When delivering a rising ramp-shaped stimulus, ASH neuron appears to show both "on" and "off" responses, of similar magnitude. (F) When the stimulus is in the shape of a decaying ramp, it leads to a strong "on" response, whereas a distinct "off" peak is absent. (G) A rising followed by a decaying ramp stimulus (triangular pulse) results in an initial "on" response, followed by a second rise during decrease of stimulus magnitude, without the characteristic "off" response peak. (H) The continuous triangular pulse in (G) can be delivered in consecutive steps. The area underneath the pulses in (G) and (H) is the same. While the overall Ca^2+^ transients in both cases are comparable, delivering the stimulus in discrete steps leads to “off” responses. Red line represents the stimulus; black line indicates the model-generated results.

We used the model to explore how the ASH neuron would respond when activated by complex time-varying stimuli that would be challenging to implement in an experimental setup, yet it is possible for the worms to encounter in nature. Fig 6 demonstrates selected examples of stimulus-evoked Ca^2+^ transients for such *in silico* experiments. In all panels, in addition to the %FRET ratio change, the Ca^2+^ concentration in the ER is also presented to show the long-lasting effects of the stimulus on the system. Compared to the changing Ca^2+^ concentration in the cytoplasm (represented by %FRET ratio change), the Ca^2+^ concentration in the ER has a slower dynamic, which affects the system’s response especially during sequential stimuli. At rest, the Ca^2+^ concentration in the ER is equal to its equilibrium value. When the stimulus is delivered, a small increase in the ER Ca^2+^ concentration is observed due to influx of Ca^2+^ through TRPVs and VGCCs. This is followed by a rapid large decrease due to Ca^2+^ release from the ER through IPRs. Then, the Ca^2+^ concentration in the ER remains relatively constant while the stimulus is sustained, since the influx and efflux of Ca^2+^ balance each other. Finally, when the stimulus is withdrawn, a second rapid large decrease in the ER Ca^2+^ concentration is observed during the “off” response. Then, the Ca^2+^ concentration in the ER starts returning to its equilibrium. However, if a stimulus is delivered before the Ca^2+^ concentration in the ER reaches its equilibrium (this is the case for all the panels in Fig 6), the dynamics of the new cytoplasmic Ca^2+^ response are different. For instance, in all cases with sequential stimuli (Fig 6A–6D), the first “on” response is the strongest. When stimuli of equal strength and different durations are applied (10, 30 and 50 sec) (Fig 6A), the magnitude for all “off” responses is almost the same. The shortest stimuli (fourth and sixth pulse, 10sec) result in just one peak. The longest stimulus (fifth pulse, 50 sec) results in a longer plateau. When we apply stimuli with the same duration but different magnitudes (2α, α, α/2, 4α) (Fig 6B), the response to the first pulse, which has twice the magnitude of the first pulse in Fig 6A, leads to stronger “on” and “off” responses. However, a stimulus with the same magnitude that is delivered later (fourth pulse) leads to weaker “on” and “off” responses.

As a next step, we applied short, repetitive (flickering) pulses (Fig 6C and 6D). When we apply a series of pulses with medium intensity (magnitude the same with the first pulse in Fig 6A) and intervals of equal duration (Fig 6C), then a strong first "on" response occurs, followed by a series of almost identical peaks, apart from the second one. No obvious difference between "on" and "off" responses is observed. Interestingly, when we implement a series of acute, very short and strong pulses (1/10 of the duration and 10 times the magnitude of the first pulse in Fig 6A), the response recorded (Fig 6D) is almost identical.

Lastly, we applied ramp pulses, where the stimulus intensity (magnitude) changes gradually over time (Fig 6E to 6G). When the pulse is in the form of a rising ramp (Fig 6E) both “on” and “off” responses are observed, but they are weaker compared to the rectangular stimulus (first pulse in Fig 6A, for example). However, for a descending ramp pulse (Fig 6F) a strong “on” response is generated (comparable to the “on” response for the first pulse in Fig 6A), without any apparent “off” response. When the pulse is triangular, namely an ascending ramp followed by a descending one (Fig 6G), the "on" response peak is like the one caused by a single rising ramp (Fig 6E). Instead of a well-defined "off" response, though, we observe a relatively mild increase of the % FRET ratio change before the signal returns to the basal level. Interestingly, when we replace the ramped triangular pulse with a stepped triangular pulse (Fig 6H), the overall shape of the change in the % FRET ratio is similar, but the system seems to respond also to the small steps, especially as the magnitude of the stimulus decreases.

## Discussion

### The dynamics of the Ca^2+^ transients mechanism is reflected in the model

TRPVs are the first channels to be activated, and the Ca^2+^ that flows in leads to IPRs opening via Ca^2+^-induced Ca^2+^ release, and to a voltage change that activates the VGCCs. The important role of TRPVs in the model is verified when they are *in silico* knocked out (Fig 2E). Neither the original model, where parameters remain as estimated before omitting a component, nor the updated model, where parameters are re-estimated after removing a component, can successfully account for the Ca^2+^ dynamics once the TRPV channels are removed.

TRPV channels are the key contributors to the cytoplasmic Ca^2+^ increase during the “on” response, even though VGCCs and IPRs contribute as well. This is mirrored in the original model, since in the absence of TRPV-mediated Ca^2+^ influx the VGCCs are not activated. Moreover, the Ca^2+^ that is released from the ER, due to IP_3_ activation via receptor-coupled EGL-30, may not be enough to trigger an "on" response.

The contribution of VGCCs to the ASH Ca^2+^ transients has been experimentally reported [4,25]. Their role in Ca^2+^ signaling in the neurons lies mainly in propagating the Ca^2+^ signal from the soma to the axon. Their contribution to the soma Ca^2+^ transients is mild [3,4]. The absence of constraints on the model parameters that control Ca^2+^ flux through these channels reflects the lack of experimental data on the relative contribution of TRPVs and VGCCs to the "on" response. In the absence of such constraints, the optimization algorithm determines the strength of each channel solely based on goodness of the fit. Thus, both the original model and the updated one can compensate for the omission of VGCCs (Fig 2F).

In contrast, the "off" response takes place even without the TRPV channels and is in fact stronger when the TRPV channels are knocked out (Fig 2E). The "off" response occurs mainly due to Ca^2+^ released from the ER, and the model takes into account the Ca^2+^-induced Ca^2+^ release, since the rates controlling the opening probability of IPRs depend on the Ca^2+^ concentration, Eqs (12)–(14). Thus, since no Ca^2+^ flows in through knocked out TRPVs during the "on" response, the Ca^2+^ release from the ER is minimized. Hence, ER stores remain full and release a high amount of Ca^2+^ into the cytoplasm, as they respond to EGL-30 mediated induction of IPRs opening during the "off" response.

In the updated model with knocked out TRPV channels (Fig 2E), the remaining parameters change, so that IPRs and VGCCs yield the "on" response even without TRPV channels. The entire Ca^2+^ response is then similar to the normal transient (Fig 2A) and it includes an "off" response. However, certain features are not accounted for, as for example the time needed for the "on" peak and the respective decaying slope, indicating that the presence of TRPVs in the model is indispensable. It is hard to imagine a real neuron capable of compensating for complete loss of TRPV channels by upregulation of VGCCs and IPRs mechanism, therefore the inability of the updated model to account for the full dynamics of the Ca^2+^ transients may reflect a real biological constrain.

At the same time, VGCCs are gated by voltage changes, which in the cell are not generated only due to the Ca^2+^ influx, but also due to other ions that enter the cytoplasm upon neuronal activation. We do not explicitly include the dynamics of each of the other ion transients in the model. However, we do include players that account for the combined effects of these ion transients. The experimental data available in the literature regarding voltage traces in the ASH neuron is limited to events related to the "on" response [4]. Therefore, we use a reduced order modeling approach to model the membrane potential to construct a phenomenological model that generates a graded voltage response (Fig 2G), which matches measurements reported for other *C*. *elegans* neurons [36–40]. Hence, our model accounts for the contribution of voltage gated channels only coarsely.

PMCA is the main ion pump responsible for removing excess Ca^2+^ from the cytoplasm in *C*. *elegans* [41]. Therefore, it is no surprise that *in silico* knockout of PMCA (Fig 2B) results in partial (original model) or complete (updated model) inability for the model to compensate for the PMCA function. We choose not to include in the model the Na^+^/Ca^2+^ exchanger, which also exports Ca^2+^ from the cytoplasm, although its presence has been reported in *C*. *elegans* [42,43].

In eukaryotic cells, there are pumps to facilitate the Ca^2+^ transport back into Ca^2+^ stores. The most prominent among them is the SERCA [44] which, depending on its isoform, is abundant on the sarcoplasmic and endoplasmic reticulum membrane of muscle cells and neuronal cells [45–48]. In *C*. *elegans*, functional SERCA homologs have been discovered [48,49]. One orthologue of SPCA, a Golgi-related Ca^2+^—transport ATPase, functioning as Ca^2+^ transporter, has also been reported [50–52]. *C*. *elegans* neurons are known to have the typical eukaryotic Ca^2+^ signaling tools [53], including functional Ca^2+^ stores. In our model, all Ca^2+^ stores are considered as one entity, depicted as ER, and the respective pumps are modeled as one mechanism, herein referred to as SERCA.

SERCA's omission from the model (Fig 2C) results in most of the ER Ca^2+^ being released without being replenished, during the “on” response. Therefore, in the original model, there is not enough Ca^2+^ left in ER to yield the “off” response. However, even without an active SERCA pump, the modeled leak across ER can restore Ca^2+^. In the updated model for SERCA knock out, the hybrid optimization algorithm takes into account that there has to be an "off‴ response upon withdrawal of the stimulus. Therefore, the remaining parameters change to save Ca^2+^ in the ER for the "off" response. This leads to forced increased influx of Ca^2+^ through TRPV channels during the “on” response. This way there is enough Ca^2+^ stored in the ER to produce the “off” response later in the updated model, even with SERCA removed.

However, *in vivo* a cell does not upregulate *a priori* the TRPV-mediated influx of Ca^2+^ upon neuronal activation to afford an "off" response later. Moreover, without a SERCA pump, the "off" response could not possibly occur in case of sequential stimuli, as replenishment of intracellular stores would not take place. Hence, it is suggested that the results of the original model illustrate better the consequences for the cell, should the SERCA pumps be impaired (Fig 2C, continuous line). This highlights the way in which the model can be used in a biologically meaningful way, to make reasonable hypotheses that may guide targeted experimental efforts.

The IP_3_ receptor, a major intracellular Ca^2+^ release channel, has been reported in *C*. *elegans* [54–56]. As shown in the original model (Fig 2D), the magnitude of the “on” response decreases dramatically when IPRs are omitted. However, the updated model parameters rescue most features of the “on” response, by increasing the input of TRPVs and VGCCs. Nevertheless, since the “off” response is attributed mainly to the release of Ca^2+^ from the ER, even the updated parameters fail to capture it. This shows that certain molecular players are irreplaceable, even when other agents are artificially asked to compensate for them.

The characteristic "off" response of the ASH Ca^2+^ dynamics, is observed also in other *C*. *elegans* sensory neurons [2,57]. However, it has not been included so far in any mathematical model for *C*. *elegans* Ca^2+^ dynamics. Elucidating the respective physiological mechanisms is work in progress [11,24]. Based on experimental results, which show that the TRP channel OSM-9 is required for the "off" response [24], and on the results of our model, we claim that the ASH “off” response is attributed mainly to the efflux of Ca^2+^ from the intracellular stores. This is an event related to OSM-9, via the Ca^2+^-induced Ca^2+^release mechanism. Therefore, the proposed model encompasses and accounts for experimentally obtained results.

Additional improvements could be applied to the model, if more experimental and electrophysiological data were available. For example, in young stressed worms the model successfully captures the overall Ca^2+^ dynamics, except for a small region right after the “on” response. One possible explanation is that, as explained, the current model accounts for VGCCs only coarsely, thus it misses some features of Ca^2+^ transients related VGCCs. This is supported by the fact that *egl-19* mutant worms have less steep decaying slope after the "on" response [25]. The models’ variations around the “off” response of aged worms may be related to the high variation observed also experimentally in the "off" response of in this age cohort ([22] S2C Fig, [19] S1 Fig).

The *in silico* knock outs are informative primarily about how the model works and illustrate its minimality and purposefulness. When it comes to explaining how the cells work, the *in silico* knock outs can serve as indications and possibly guides to targeted experiments.

### Ca^2+^ dynamics reveal the effect of age and stress on Ca^2+^ signaling machinery

The overall ability of our model to capture the Ca^2+^ dynamics in all four worm groups studied is excellent (Figs 2A, 3A and 3B). The model accounts for variability in the experimental data, as shown in the sensitivity analysis (Fig 5) and shows the same variation observed in the experimental data, when the model parameters are perturbed. Yet, the model is robust enough not to lead to unreliable results away from what is experimentally anticipated. Potential intercellular interactions between ASH and other neurons are not considered in the present model. It is possible that ASH might be affected by oxidative stress- or aging-induced changes in other neurons. The proposed effects intend to offer plausible explanations regarding intracellular events.

There are more than one parameter sets that can equally explain the changes in Ca^2+^ dynamics. This is because of multiple possible ways that can lead to similar cell responses. The correlation between different channels in neurons that produce similar electrical properties has already been established [58]. Our model reflects similar phenomena. For instance, the elevated Ca^2+^ response due to aging may occur because PMCA is weakened or because TRPVs are more sensitive or perhaps due to a combination of the two. In fact, even though the effect of aging on the Ca^2+^ response is approximately the same among worms of the same age, their biological cause may be different among individuals. As expected, such correlations introduce difficulties when searching for a unique parameter set in the model.

Each parameter may affect the outcome of the model in more than one ways. For example, *G*_*IPR*_ affects both the "on" and "off" responses (Fig 1D), and *G*_*PMCA*_ affects the decaying slope of both responses, and also the magnitude of the “on” response (Fig 2B). Consequently, quantitative correlations of parameter values exclusive to one specific feature of the Ca^2+^ transient are precarious. However, qualitative connections between parameters and physiological changes in the Ca^2+^ transients can be articulated, in combination with experimental results. It is noted that worms of Day 5 of their adult life used here are considered to be middle aged; therefore the effect of aging on their Ca^2+^ transients is expected to be mild [59].

G-protein coupled receptors (GPCRs), like the ones activated by hyperosmotic stimulus in the ASH neuron [60,61], can be dysregulated by increased production of ROS [62,63]. The effect of aging on GPCRs depends strongly on how molecules related to them, especially kinases and G-proteins, are affected [64–66]. In our experimental data (Fig 3), aging affects the rising and decaying slope of both "on" and "off" responses, the latter in combination with stress. The slopes in the model could be correlated to the rates (i.e. *P*_0_, $k_{P_{0}}$, $k_{P_{0}}^{-}$) of events that involve the receptor-coupled G proteins. Moreover, oxidative stress results in faster "on" peak in aged worms. This could potentially be linked to the rates of cascades related to receptor-coupled proteins (i.e. $k_{P_{0}}$, $k_{P_{0}}^{-}$). These rates ($k_{P_{0}}$, $k_{P_{0}}^{-}$) are included in the model’s plausible solutions for young stressed and aged unstressed worms (Fig 4A, 4B, 4C and 4D). Therefore, the model can assist on choosing potential experimental targets, to explore the dynamics of biological events.

Although TRP channels are involved in lifespan regulation in *C*. *elegans* [67], changes in TRPV activation with respect to aging have not been reported yet. Studies in mammalian systems indicate that expression or distribution of TRPV channels is affected by aging [68,69]. Our experiments show that the magnitude of the "on" and the "off" responses increases with aging (Fig 3C and 3D top panels). This could be related to changes in the probability of TRPVs being activated (parameters *k*_*O*_ and $k_{O}^{-}$) in aged worms (Fig 4E and 4F) as well as to changed rates for the G protein related cascades ($k_{P_{0}}$, $k_{P_{0}}^{-}$) (Fig 4C and 4D). Thus, the model can suggest potential roles for molecular players involved in aging-driven changes in the Ca^2+^ transients.

Oxidative stress has a significant impact on TRPV channels, mainly through oxidation of cysteine residues in channels' subunits [70,71], resulting in TRPV sensitization [70,72]. Experiments show that oxidative stress affects the magnitude and rising slope of the "on" response in aged worms, and the magnitude of "off" response in younger worms (Fig 3). These features can be linked to changes in the rates for the G protein related cascades ($k_{P_{0}}$, $k_{P_{0}}^{-}$, in young worms) or the probability of activated TRPV channels (*k*_*O*_ and $k_{O}^{-}$, in aged worms, Fig 4C, 4D, 4E and 4F). Therefore, the results of our model corroborate experimental findings in *C*. *elegans* and other systems.

VGCCs are known to be affected by oxidative stress [73] via oxidation of cysteine residues [74] and–SH groups [75]. In aged neurons, VGCCs show increased activity [76,77], and their contribution to Ca^2+^ influx rises in both cases [76,78]. Interestingly, we do not detect any significant change regarding VGCC channels in our model. This could be because VGCCs contribute less than TRPVs to Ca^2+^ transients in the neuronal body, where our efforts are focused. Moreover, VGCCs are modeled coarsely in the present work, not allowing us to draw solid conclusions.

The effect of aging on neuronal IP_3_ receptors [78] results in elevated flux of Ca^2+^ into the cytoplasm [79,80], partially through enhanced Ca^2+^-induced Ca^2+^ release [81]. Age increases the rising and decaying slopes of the "on" and "off" responses in our experiments, the latter only in stressed animals (Fig 3C). The slopes in the model may be related to the maximum ion flux though IPRs (*G*_*IPR*_), and to the rates for IPRs (*k*_*p*_ and $k_{p}^{-}$). These parameters are indeed included in the plausible solutions for aged and for stressed worms (Fig 4C to 4F). This indicates that the model can successfully account for experimentally detected physiological changes.

Oxidative stress leads to increased Ca^2+^ efflux from the stores into the cytoplasm [82,83] through IP_3_ receptors, because of–SH groups oxidation [80] and increased production of IP_3_ [82]. Experiments show that oxidative stress affects the magnitude and rising slope of "on" response in aged worms, and the "off" response peak in young worms (Fig 3C and 3D). The responses magnitude can be linked to the maximum ion flux through IPRs (*G*_*IPR*_), which changes in stressed worms (Fig 4A and 4B). Oxidative stress also results in faster "off" peak in young worms (Fig 3F), which could be related to altered maximum ion flux through IPRs (*G*_*IPR*_) and to the rates for IPRs (*k*_*p*_ and $k_{p}^{-}$) (Fig 4A and 4B). Hence, the model accounts satisfactorily for the role of intracellular Ca^2+^ channels described by experimental data.

In contrast to the increased activity of ion channels due to oxidative stress, the PMCA pump is inactivated by ROS [82], as a result of altered tyrosine and methionine residues [84]. Decreased efflux of Ca^2+^ through PMCAs occurs also in aged neurons [85,86]. SERCA pumps are affected in a similar way, since they are inhibited by oxidative stress [87] and are also impaired in aged neurons [88]. The magnitude of the "on" and the "off" response increases with aging (Fig 3C) in *C*. *elegans*. This could be related to changed affinity of PMCAs and SERCAs for Ca^2+^ (*K*_*PMCA*_ and *K*_*SERCA*_) in aged worms (Fig 4C to 4F). Experiments show also that oxidative stress affects the peak and rising slope of the "on" response (Fig 3C and 3D) in aged worms, and of the "off" response in young worms (Fig 3C). The response magnitude can be linked to the changed affinity for Ca^2+^ of SERCAs (*K*_*SERCA*_) in young stressed worms (Fig 4A and 4B). Stress results in altered decaying slope after the "on" and "off" response of young worms (Fig 3E), which could possibly be related to changed maximum ion flux through PMCAs (*G*_*PMCA*_), as is suggested in the model (Fig 4A and 4B). Therefore, the way aging and oxidative stress affect the function of major cell pumps is well captured by the proposed model.

To summarize, our model can be used to explore the effects of aging and stress on the ASH Ca^2+^ signaling machinery. The suggested correlations between the effect of aging and oxidative stress on physiological cascades and the model parameters are not the only possible explanation for the observed changes in Ca^2+^ dynamics and should be confirmed experimentally. Parameters that are not included in the plausible solutions may also play a role. However, the agreement of the model with the available experimental data strongly suggests that the model and the selected parameters capture successfully the physiological trend.

### The model can predict complex Ca^2+^ transients

*C*. *elegans* sensory neurons respond differently to varying stimulus strengths [89]. Our model generates Ca^2+^ transients peaks of different magnitudes in ASH neuron, in both "on" and "off" responses, depending on the strength of the applied stimulus (Fig 6B). However, hidden states with slower dynamics, i.e. ER Ca^2+^ dynamics, that are not easily measured in experiments but are captured by the mathematical model, may have an important impact (Fig 6B, differences in sequential responses).

The response to gradient stimuli has been shown experimentally for *C*. *elegans* sensory neurons [89], especially for odor gradients [28,90,91]. The model-generated Ca^2+^ transients that occur in the presence of a ramp stimulus (Fig 6E, 6F and 6G) indicate that the ASH neuron can detect smooth gradients via the "on" response and sharp decreases via the “off” response. Therefore, our model suggests that the “off” response may be a mechanism that detects acute changes.

A step-increasing pulse, instead of a continuous gradient, (Fig 6G and 6H) does not affect significantly the overall shape of the Ca^2+^ transient (the area underneath both pulses is the same). However, the decreasing stimulus through stepped pulses results in different “off” responses, as has been reported experimentally (33). This suggests that the proposed model can encapsulate Ca^2+^ dynamics in the ASH neuron in alignment with the experimental data.

The ability of ASH to respond reliably to a sequence of identical stimuli has been shown experimentally [25], and interestingly, the neuron responds consistently, especially via the "off" response (S5 Fig of [25]). Indeed, when arrays of sequential pulses are applied in the model, results capture the "off" response robustness (Fig 6A and 6B).

In our experiments, the duration of the stimulus is 30 sec [22], as in other similar studies [18,19]. Previous work (18) suggests that a shorter hyperosmotic stimulus (15 sec) leads to a weaker “on” response and a stronger “off” response, compared to a long stimulus delivery in the same study. When a short stimulus is applied in our model (Figure A in S1 Fig) the "off" peak is milder than the "on". We hypothesize that the stronger “off” response reported in (18) is a consequence of the weaker “on” response and not of the short stimulus duration. To test this hypothesis, we tried different values for the *K*_*c*_ parameter in Eq (16), which affects the dynamics of IP_3_ production, to change the magnitude of the “on” response (S1 Fig). Indeed, a weaker “on” response leads to a stronger “off” response, without changing the duration of the stimulus, possibly due to full intracellular Ca^2+^stores. In the paper by Chronis and colleagues [19], the reason behind the shape of the Ca^2+^ transient of the 15 sec pulse could be the small population examined (3 traces, Suppl. Data [19]). Therefore, our model can help interpret experimental variations in *C*. *elegans* populations.

Kato and colleagues [25], showed that the ASH neuron can respond with precision, through its Ca^2+^ transients, to flickering stimuli of 1 sec long pulses (Fig 1 of [25]). Our model can capture such a flickering response (Fig 6C and 6D). Therefore, the model can be a reliable computational tool when exploring the response of *C*. *elegans* sensory neurons. Moreover, the model shows that the stimulus-evoked Ca^2+^ transient is the same when the total amount of stimulus-carrying solution delivered remains the same (as indicated by the area under each pulse) and does not depend solely on the actual duration or magnitude of each pulse (Fig 6C: 2units of stimulus/5sec; Fig 6D: 10units of stimulus/1sec). This is an interesting finding, which can elucidate the details of the ASH neuron activation mechanism, if corroborated by experimental data.

The fast responses of the system (Fig 6D and 6C) captured by our model require further validation, because the equations of the model have been developed based on slower dynamics (Fig 3A and 3B). However, the capability of the proposed model to successfully account for *in silico* trials shows that it can serve as a preliminary tool for pre-experimental tests.

## Conclusions

We propose a model for the Ca^2+^ dynamics in the *C*. *elegans* ASH polymodal neuron, based on intracellular events that unfold as part of the Ca^2+^ signaling machinery. Our model uses experimental data on stimulus-evoked Ca^2+^ transients upon neuronal activation and it captures efficiently for the first time the dynamics of both the “on” and “off” responses. Moreover, the model can account for changes in the ASH Ca^2+^ dynamics due to age and exposure to oxidative stress, reflecting, confirming and sometimes predicting the role of each molecular player modeled in the cellular mechanism that generates Ca^2+^ transients. We believe that the proposed model makes a contribution in *C*. *elegans* neurobiology because it i) suggests how molecular players that are known to be affected by aging and/or oxidative stress may be involved in generating the altered Ca^2+^ transients features (e.g. IP_3_ receptors), ii) suggests a possible effect of aging and/or oxidative stress on molecular players not yet documented to play a role toward aging- or stress-driven modifications of the Ca^2+^ transients features (e.g. activation/inactivation of TRPV channels), and iii) can be used to propose and guide future experimental work, targeting specific molecular players involved in Ca^2+^ dynamics. Finally, the model can be used to predict Ca^2+^ transients in the ASH neuron in the case of complex stimuli. We envision the proposed model as a tool used to further elucidate the Ca^2+^ dynamics of *C*. *elegans* neurons and to pave the way for the development of more mathematical models for neuronal Ca^2+^ dynamics.

## Methods

The model is based on intracellular molecular events responsible for the generation of Ca^2+^ transients. Numerous molecules and pathways are involved in this process [92,93]. We focused on molecular players which are believed to dominate the dynamics [4,25,77,79–81], taking also into account that some of the remaining molecular players are not yet well understood in *C*. *elegans* neurons [78]. We modeled the dynamics of the secondary players using a coarse grain approach, similar to reduced order modeling. To this end, we introduced selected equivalent players (viewed as states in the model) whose dynamics captures the overall combined effects of most of the secondary players.

### Mathematical model of Ca^2+^ dynamics

Assuming a cell with well-mixed free Ca^2+^, the concentration of free Ca^2+^ in the cytoplasm and endoplasmic reticulum (ER) can be written as the following system of ordinary differential equations [94]:

$$\frac{dc}{dt}=J_{IPR}+J_{Leak,ER}-J_{PMCA}-J_{SERCA}+J_{TRPV}+J_{VGCC}+J_{Leak},$$ (1)

$$\frac{dc_{ER}}{dt}=\gamma (J_{SERCA}-(J_{IPR}+J_{Leak,ER})),$$ (2)

where *c* and *c*_*ER*_ are the concentrations of free Ca^2+^ in the cytoplasm and in the ER. *J*_*PMCA*_ represents the Ca^2+^ efflux through plasma membrane Ca^2+^ ATPase (PMCA) pumps to the extracellular space (ES), and *J*_*SERCA*_ is the Ca^2+^ flux from the cytoplasm into ER through ER ATPase pumps. *J*_*TRPV*_ and *J*_*VGCC*_ denote the influxes of Ca^2+^ into cytoplasm from ES through transient receptor potential-vallinoid (TRPV) channels and through voltage gated Ca^2+^ channels (VGCC). *J*_*Leak*_ and *J*_*Leak*,*ER*_ represent constant influxes of Ca^2+^ into the cytoplasm from ES and ER through other mechanisms. *γ* denotes the ratio of the cytoplasmic volume to the ER volume, which can also account for fast linear Ca^2+^ buffers in the ER [95].

The PMCA pumps are modeled following [94] as:

$$J_{PMCA}=G_{PMCA}\frac{c^{2}}{c^{2}+K_{PMCA}},$$ (3)

$$J_{SERCA}=G_{SERCA}\frac{c^{2}}{c^{2}+K_{SERCA}},$$ (4)

where *G*_*PMCA*_ and *G*_*SERCA*_ are the maximum fluxes through PMCA and SERCA pumps. *K*_*PMCA*_ and *K*_*SERCA*_ model the affinity of PMCA and SERCA for Ca^2+^. Ca^2+^ in the cytoplasm and the ER is always at equilibrium before applying the stimulus. To that end, the leak parameters across the cell membrane and the ER need to be adjusted based on the parameter values for PMCA and SERCA, and on the initial concentration of Ca^2+^ in the cell and the ER. The necessary leak values for equilibrium can be found by setting the time derivatives in Eqs 1 and 2 (for example, when PMCA is knocked out *in silico*, the leak across the membrane will be found equal to zero).

Even though TRP channels show weak voltage dependence [96], due to limited evidence regarding TRP voltage sensitivity in *C*. *elegans* neurons, this behavior is not included in the model. The Ca^2+^ influx through TRPV channels is modeled as:

$$J_{TRPV}=G_{TRPV}O.I(c_{ES}-c),$$ (5)

where *G*_*TRPV*_ is the maximum influx of Ca^2+^ through TRPV channels, and *c*_*ES*_ denotes the Ca^2+^ concentration in the ES, which is assumed to be constant and *c*_*ES*_ = 2000 μM. *O* and *I* are the probabilities of TRPV channels to be activated and inactivated, and are governed by:

$$\frac{dO}{dt}=k_{O}P_{1}(1-O)-k_{O}^{-}O,$$ (6)

$$\frac{dI}{dt}=k_{I}(1-I)-k_{I}^{-}P_{2}I,$$ (7)

where *k*_*O*_, *k*_*I*_ are forward and $k_{O}^{-}$, $k_{I}^{-}$ are backward rate constants for the *O* and *I* states. *P*_1_ and *P*_2_ represent two molecular players that control activation and inactivation of TRPV channels with the following kinetics:

$$\frac{dP_{1}}{dt}=k_{P_{1}}P_{0}-k_{P_{1}}^{-}P_{1},$$ (8)

$$\frac{dP_{2}}{dt}=k_{P_{2}}\frac{P_{0}}{P_{0}+K_{P_{2}}}-k_{P_{2}}^{-}P_{2},$$ (9)

where $k_{P_{1}}$, $k_{P_{2}}$ are forward and $k_{P_{1}}^{-}$, $k_{P_{2}}^{-}$ are backward rate constants for *P*_1_ and *P*_2_, and $K_{P_{2}}$ is the affinity of *P*_2_ for an equivalent player *P*_0_ that coarsely represents the dynamic effects of the cascade of molecular players that are activated by the external stimulus. We model the dynamics of *P*_0_ as:

$$\frac{dP_{0}}{dt}=k_{P_{0}}\frac{S}{S+K_{P_{0}}}-k_{P_{0}}^{-}P_{0},$$ (10)

where *S* represents the strength of the stimulus, $K_{P_{0}}$ is the affinity of *P*_0_ for *S*, and $k_{P_{0}}$, $k_{P_{0}}^{-}$ are forward and backward rates for *P*_0_.

Player *P*_0_ coarsely accounts for the pathway that is triggered upon delivery of the stimulus, including the G-proteins which are coupled with the receptors [97,98] (ODR-3, EGL-30, Fig 1). Player *P*_1_ coarsely represents the pathway downstream of EGL-30, which leads to activation of TRPV channels [97]. Player *P*_2_ represents the cascade of events in which fatty acids participate, modulating TRPV channels [93,99] (Fig 1).

Experimental results (10, 23, 24) show that a stronger stimulus leads to a stronger Ca^2+^ response in ASH neuron, although this finding has been challenged [4,8]. Therefore, *P*_0_, which in our model translates the stimulus input into sparking molecular cascades, increases with the strength *S* of the stimulus. However, the dependence of *P*_0_ on S is not linear, to allow saturation of *P*_0_ for very strong stimuli and reflect the reality that an infinitely strong response is not possible. Both *P*_1_ and *P*_2_ are promoted by *P*_0_. Hence, a stronger stimulus leads to a larger *P*_0_, which in turn produces larger *P*_1_ and *P*_2_. Larger *P*_1_ leads to stronger activation of TRPV channels, while larger *P*_2_ leads to stronger inactivation of TRPV channels. Activation needs to surpass inactivation (*P*_1_ must respond more strongly to the stimulus compared to *P*_2_) to observe a stronger Ca^2+^ influx when a stronger stimulus is applied, as dictated by the underlying assumption (10, 23, 24). Therefore, *P*_2_ in our model saturates with *P*_0_ (see Eq 9) to allow for stronger activation with stronger stimulus.

The Ca^2+^ influx through IP_3_ receptors (IP_3_Rs) is modeled as:

$$J_{IPR}=G_{IPR}O_{IPR}(c-c_{ER}),$$ (11)

where *G*_*IPR*_ is the maximum Ca^2+^ flux through IP_3_ receptors, and *O*_*IPR*_ is the probability of IP_3_ receptors (IPRs) to be open, which is modeled using a reduced form [100,101] in the De Young-Keizer model [102], namely:

$$O_{IPR}={\left(\frac{pc(1-y)}{\left(p+K_{1})(c+K_{5}\right)}\right)}^{3},$$ (12)

where *p* is the concentration of IP_3_, and *y* is the faction of inhibited IPRs. The faction of inhibited IPRs is in turn governed by:

$$\frac{dy}{dt}=\phi_{1}(1-y)-\phi_{2}y,$$ (13)

where

$$\phi_{1}=k_{2}^{-}c\frac{k_{42}K_{2}K_{1}+K_{4}p}{K_{4}K_{2}(K_{1}+p)},$$ (14)

$$\phi_{2}=k_{2}^{-}\frac{p+k_{42}K_{3}}{K_{3}+p},$$ (15)

with $K_{i}=k_{i}^{-}/k_{i}^{+}$ being the equilibrium constants for binding/unbinding of IP_3_ or Ca^2+^ to IPRs, with their original values, as used by De Young and Keizer [102]. It is not clarified which phospholipase-C (PLC) isoform is present in ASH neuron. To this end, we have not included in the model any dependence of IP3 on PLC.

To model variations in *p* upon the delivery of the stimulus, we use players *P*_1_ and *P*_2_ together with *c* to create the following phenomenological model:

$$\frac{dp}{dt}=k_{p}\frac{P_{1}}{1+{\left(K_{p}P_{2}\right)}^{4}}\frac{c^{2}}{c^{2}+K_{c}^{2}}-k_{p}^{-}p,$$ (16)

where *k*_*p*_ and $k_{p}^{-}$ are constants that represent forward and backward rates, while *K*_*p*_ and *K*_*c*_ are the affinities of *p* for *P*_2_ and *c*. The relation between *P*_1_ and *P*_2_ in Eq (16) follows an incoherent feed-forward network motif [103], which is also combined with a Hill equation [94] for Ca^2+^. This part of the model plays an important role in the "off" response. IPRs are responsible for the release of Ca^2+^ from intracellular stores. IP3 concentration (*p*) is the only free parameter in the original model of IPRs dynamics [102]. One option is to introduce new molecular players to govern the dynamics of *p*. However, that approach adds states to the model. Instead, *P*_1_ and *P*_2_ (two molecular players already present in the model) are used to capture the dynamics of *p*. Players *P*_1_ and *P*_2_ coordinate the dynamics of *p* in a way that the surge of p occurs when the stimulus is withdrawn, to allow release of Ca^2+^ from stores. Such dynamics for *p* can be achieved using incoherent feed-forward motifs [103] for generation of *p* by *P*_1_ and *P*_2_. Moreover, the term in Eq 16 that depends on *c* (Ca^2+^ concentration) accounts for the generation of *p*. This term saturates when the concentration of Ca^2+^ increases, to incorporate in the model the well-known phenomenon of Ca^2+^-induced Ca^2+^ release from intracellular stores [104].

The Ca^2+^ influx through L-type voltage gated calcium channels (VGCCs) are modeled using the Goldman-Hodgkin-Katz [105] equation:

$$J_{VGCC}=G_{VGCC}m_{\infty }^{2}V\frac{c-c_{ES}e^{\frac{-zFV}{RT}}}{1-e^{\frac{-zFV}{RT}}},$$ (17)

where *G*_*VGCC*_ is the maximum Ca^2+^ flux through VGCC, *z* is the valence of the respective ions (*z* = 2 for Ca^2+^), *F* is the Faraday constant, *R* is the gas constant, *T* is the temperature, *V* is the membrane voltage, and *m* is the activation variable given by:

$$m_{\infty }(V)=\frac{\alpha (V)}{\alpha (V)+\beta (V)},$$ (18)

where *α* and *β* are rates that vary with the membrane voltage for the L-type current as follows [106]:

$$\alpha (V)=\frac{1.6}{1+e^{-0.072(V-5)}},$$ (19)

$$\beta (V)=\frac{0.02(V-1.31)}{e^{(V-1.31)/5.36}-1}.$$ (20)

Finally, we model the dynamics of membrane potential *V*. Unfortunately, the available experimental data regarding changes in the membrane potential of the *C*. *elegans* ASH neuron is limited. Moreover, the ion currents, which are responsible for the voltage response of *C*. *elegans* neurons, are not completely understood. However, the available data suggests that many *C*. *elegans* neurons show a graded voltage response rather than the (better characterized and more commonly encountered) action potential response [36,39,40]. Therefore, we use a phenomenological approach to model a graded voltage response based on the only ionic current, namely the Ca^2+^ current, as follows:

$$V=\alpha \prime (V_{max}-V)-\beta \prime (V-V_{rest}),$$ (21)

where *V*_*max*_ is the maximum membrane potential that can be reached during the graded response of the ASH neuron, and *V*_*rest*_ = −70mV is the resting membrane potential. *α*′and *β*′ are forward and backward rates for Eq (21), and are given by:

$$\alpha^{\prime }(c)=\frac{-26c+4}{e^{-26c+4}-1}.$$ (22)

$$\beta^{\prime }(c)=\frac{260c-20}{e^{13c-1}-1}.$$ (23)

Note that *α*′ and *β*′ are similar to *α* and *β* in Eqs (19) and (20). However, they have different values, and are functions of *c* (concentration of free Ca^2+^ in the cytoplasm) instead of *V*.

### Ca^2+^ concentration to FRET signal conversion

The Ca^2+^ concentration obtained using the mathematical model can be related to signals measured in experiments [18,22]. Experimental signals obtained using TN-XL FRET (fluorescence resonance energy transfer) measurements [107] are the result of Ca^2+^ interaction with an indicator genetically encoded in the *C*. *elegans* ASH neuron. The relation between the Ca^2+^ concentration and the measured % FRET change signal *R* follows an empirical form [108] that can be expressed as:

$$\begin{array}{l} \Delta R\%=\frac{R-R_{min}}{R_{max}-R_{min}}=R_{max}\frac{c^{n}}{c^{n}+K_{d}^{\prime n}},\\ FRET=\frac{R-R_{0}}{R_{0}}\times 100, \end{array}$$ (24)

where the Hill coefficient is *n* = 1.7, and the apparent affinity of *R* for Ca^2+^ is $K_{d}^{\prime n}=2.5$, as provided in [107].

Eq (24) can be used to convert measured % FRET changes into the corresponding Ca^2+^ concentration. The use of Eq (24) a stronger stimulus leads of *R*_*min*_, *R*_*max*_ and *R*_0_. To this end, we used the data in [108,109] to set the baseline Ca^2+^ concentration to 100nM. Also, we set R = 5 in Eq (24) for the baseline Ca^2+^ concentration [S2 Fig of ref 22]. We set FRET ratio change = 10% when Ca^2+^ concentration equals 500nM [110]. Hence, we calculate *R*_*min*_ and *R*_*max*_ based on the Ca^2+^ that we set. set the baseline Ca^2+^ concentration to 100nM and the maximum Ca^2+^ concentration to 500nM when Δ*R*% = 10 (for young unstressed worms), and then calculated *R*_*min*_ and *R*_*max*_. Next, we determined *R*_0_ using Eq (24) based on the initial value of the Ca^2+^ concentration in the mathematical model, which is set to 100nM for all cases.

### Parameter estimation

There are 23 parameters (S1 Table) that need to be determined in order to use the mathematical model proposed. Ultimately, the mathematical model must capture the Ca^2+^ dynamics in four experimental data sets that are used in this study, namely measured Ca^2+^ transients for young unstressed, young stressed, aged unstressed and aged stressed worms. We chose the experimental data set for young unstressed worms as reference to determine all 23 parameters. Next, we modified the values of as few of these parameters as necessary to fit the data for the other three cases, one at a time. This approach allows us to suggest different possible pathways through which the parameters governing Ca^2+^ transients may change, to capture alterations in stimulus-evoked Ca^2+^ dynamics in aged worms or in worms previously exposed to oxidative stress.

We used a hybrid optimization approach, which consists of a genetic algorithm (GA) as a global minimizer, and trust-region-reflective nonlinear least squares (TRNLS) as a local optimizer, to determine the parameters of the mathematical model. The hybrid optimization algorithm starts with the GA, and the fittest individual found from the GA is passed to the TRNLS. The minimization problem is also subjected to constraints that ensure all parameter values are physical (namely that they are positive). The MATLAB optimization toolbox is used to perform these calculations. The maximum-likelihood of the experimental data is the objective function used in the TRNLS algorithm, namely:

$$Res=\frac{FRET_{model}-\mu_{exp}}{\sigma_{exp}},$$ (25)

where *Res* is the residual at each measurement instant, *FRET*_*model*_ is the data obtained from the model, *μ*_*exp*_ and *σ*_*exp*_ are the average and the standard deviation of experimental data at that same time instant. The objective function to be minimized in the GA is the sum of all residuals given by Eq (25) over all time instants measured.

We determined first the parameters for young unstressed worms. The solution of the GA depends on the initial population. Thus, we first applied the GA as a multi-objective optimizer to all four sets of experimental data. We then used the resulting population as an initial population for the GA applied only to the data from the young unstressed worms. Among the optimum solutions suggested by the multi-objective GA, we chose the solution for which the sum of the residuals for all experimental cases was the minimum, even though there were other solutions in which the residual for individual cases was smaller than the chosen solution. This approach allowed us to select as initial population for the GA a vicinity in the parameter space that is near an optimum solution (minimum residual) for all four cases.

There are more than one parameter sets that can result in the measured Ca^2+^ dynamics for young unstressed worms. However, we sought a single set of parameters for young unstressed worms for which the sensitivity of the solution for the estimated parameters was small. To that end, we used the numerical Jacobian matrix that is obtained from TRNLS to approximate the Hessian matrix. Then, we used the Hessian matrix to construct the covariance matrix. We chose the solution that had the smallest diagonal elements of the covariance matrix. That corresponds to choosing the solution with the smallest variance of the parameters, indicating that small changes in the parameters (for that solution) do not lead to vast changes in the Ca^2+^ dynamics.

For the other three experimental cases, we start with the parameters found for young unstressed worms to create the initial population for the GA in the hybrid optimization. Next, we limit the algorithm to change only 13 selected parameters of the total of 23. These 13 parameters (listed with bold in S1 Table) include strength and rates related to key players of the Ca^2+^ signaling mechanism–TRPV channels being activated/deactivated, IP_3_, IPRs, PMCAs, SERCAs–and they have been selected based on discussions in the existing literature [55,111–113]. Then, we use the hybrid optimization algorithm to estimate the values of the 13 selected parameters, while the other parameters are kept constant. A similar procedure is performed separately for all possible combinations for the selected parameters. Next, the results from all the combinations are pooled together for each of the three experimental cases. Each combination of selected parameters is a potential pathway that can show effects of aging or oxidative stress on young unstressed worms. However, not all the combinations are plausible. The first criterion used to select valid combinations of parameter sets, is the goodness of fit that the mathematical model provides, i.e. the residuals must be small. To make the comparison consistent among different cases, we sort the residual for all different combinations and only keep the combinations for which the residuals are smaller than 99% of all solutions. Next, we use a second criterion on the remaining combinations in which parameter changes that are detectable are selected. To that end, we compute changes in parameters as compared to young unstressed worms. If the absolute value of the changes for a parameter combination is larger than the sensitivity found using the covariance matrix, then we consider such combination plausible.

A component with more than one values for its parameters is still necessary in the model, even though different parameter sets can lead to same qualitative results. For instance, different combinations of parameters for PMCA and TRPV channels can provide relatively similar results. However, including PMCA cannot be claimed to be redundant just because several values can be suggested for it as the model breaks without PMCA (Fig 2B).

#### Ca^2+^ transients experimental data analysis

All experimental results presented in Figs 2 and 3 were acquired as described in Gourgou and Chronis, 2016 [22]. Briefly, using the TN-XL FRET sensor, the stimulus-evoked Ca^2+^ transients generated when the ASH neuron of stressed (exposed to oxidative stress) and unstressed *C*. *elegans* of various ages was stimulated by hyperosmotic solution of 1M glycerol [18,22] were recorded [22]. The FRET signal change was recorded in the microfluidic device [35], where each worm was introduced after growing in the presence of the oxidative stress-causing chemical paraquat [35]. Experimental data used in this study come from four populations of adult hermaphrodite *C*. *elegans*: i) young (L4+1/Day 1 of adult life) unstressed animals (control animals; reference case), ii) young (L4+1/Day 1 of adult life) oxidative-stressed animals, iii) aged (L4+5/Day 5 of adult life) unstressed animals, and iii) aged (L4+5/Day 5 of adult life) oxidative-stressed animals. It is noted that Day 5 worms can be considered of middle age [114], but for brevity they are referred to as the aged worms.

The peak and the slope of the rising phase of the "on" response in the ratio traces were calculated at the onset of stimulus (Fig 3C, top; see also Fig 1B of Gourgou and Chronis, 2016[22]). The peak of the rising phase is indicative of the total amount of Ca^2+^ entering the cell and consequently accounts for the amplitude of the cell's response to the applied stimulus, whereas the slope corresponds to the time rate of the Ca^2+^ influx.

Here, we calculate also the decaying slope of the "on" response (Fig 3E, top), which corresponds to the time rate of the Ca^2+^ efflux off the cytoplasm that takes place after the neuron's initial response to the applied stimulus and leads to a stabilization (plateau) of the cytoplasmic Ca^2+^ after 30 sec of the response onset and until the stimulus is withdrawn. The maximum peak of the "off" response (Fig 3C, bottom) is indicative of the total amount of Ca^2+^ entering the cytoplasm upon withdrawal of the stimulus. The rising and descending slopes of the "off" response (Fig 3D and 3E, bottom panels) account for the time rate of Ca^2+^ influx to the cytoplasm and the time rate of Ca^2+^ efflux off the cytoplasm, both upon withdrawal of the stimulus.

The decaying slope of the "on" response can be defined as the difference between the maximum value of the FRET ratio change on_*max*_ observed upon delivery of the stimulus and the average stabilized FRET signal A_*plateau*_ at the plateau between 30 and 40 sec, over the time needed for this decay, namely (on_*max*_ − *A*_*plateau*_)/(*T*_*minplateau*_ − *T*_*max*_), where *T*_*max*_ is the time when on_*max*_ is observed, and *T*_*minplateau*_ is the time when the plateau starts.

The maximum peak off_*max*_ of the "off" response is calculated as the % peak FRET ratio change upon withdrawal of the stimulus. The rising slope of the "off" response is the difference between the average stabilized FRET signal A_*plateau*_ at the plateau between 30 and 40 sec and the maximum peak off_*max*_ observed upon withdrawal of the stimulus, over the time needed to reach this maximum, counting from the stimulus offset. The rising slope can be expressed as (off_*max*_ − *A*_*plateau*_)/(*T*_*max*_ − 40).

The decaying slope of the "off" response is the maximum value of the % FRET ratio change off_*max*_ observed upon withdrawal of the stimulus over the time needed to reach the minimum value observed after the stimulus offset, namely off_*max*_/(*T*_*offmax*_ − *T*_*min*_).

#### Statistical analyses

All comparisons in Fig 3 are made using two tailed, unpaired Student's *t*-test. Statistically significant differences were considered the ones with *p*-value < 0.05. Exact *p*-values are provided for each comparison on the respective plot. Statistical analyses were performed in Excel (Microsoft, WA, USA) and Minitab (Minitab Inc., PA, USA).

## Supporting information

S1 Fig The model generated Ca^2+^ transient, induced by square pulses of different durations and same strength. (A) A short pulse of 10sec still results in distinct peaks for “on” and “off” responses of different magnitudes, without the plateau region; (B) The Ca^2+^ transient induced by the pulse (30sec) delivered in the experimental data and the model results, presented here for comparison; (C) A long pulse of 50 sec results in a Ca^2+^ transient of similar shape with the one shown in (B). The response to the shorter stimulus in (A) includes an “off” response stronger than the one observed in (B) and (C), although still smaller than the “on” peak. All three Ca^2+^ transients are generated using the parameters estimated for young unstressed worms (reference case).
(PPTX)

S2 Fig The parameters of the mathematical model can be modified to capture variations in the transients that are observed experimentally. K_c_ in Eq 16, which affects dynamics of IP3, can be changed to control the relative magnitude of “on” and “off” response. A weaker “on” response leads to a stronger “off” response because when less Ca^2+^ is released from the ER during the “on” response, then there is more available to be released from ER during the “off” response. K_c_* corresponds to the value of this parameter used in the model for young unstressed worms (reference case).
(PPTX)

S1 Table Parameters descriptions, references, and values for young (Day 1) unstressed worms (reference case), as generated by the hybrid optimization algorithm. With bold are the selected parameters which are investigated for the aging and oxidative stress effect in the next three worm populations.
(PPTX)

S2 Table Parameters values for young (Day 1) stressed worms, as generated by the hybrid optimization algorithm. For each parameter, are indicated the mean of the values it takes in all sets of plausible solutions in which it appears, standard deviation, the difference of the mean with the parameter value for young unstressed worms (S1 Table), the % mean difference with young unstressed worms, and in how many of the plausible solutions for the specific worm population this parameter appears. With bold are the parameters which seem to be more important for the changes in Ca^2+^ transients in young (Day 1) stressed worms compared to the reference case, either due to their change compared to the reference case, or due to their abundance in the plausible solutions.
(PPTX)

S3 Table Parameters values for aged (Day 5) unstressed worms, as generated by the hybrid optimization algorithm. For each parameter, are indicated the mean of the values it takes in all sets of plausible solutions in which it appears, standard deviation, the difference of the mean with the parameter value for young unstressed worms (S1 Table), the % mean difference with young unstressed worms, and in how many of the plausible solutions for the specific worm population this parameter appears. With bold are the parameters which seem to be more important for the changes in Ca^2+^ transients in aged (Day 5) unstressed worms compared to the reference case, either due to their change compared to the reference case, or due to their abundance in the plausible solutions.
(PPTX)

S4 Table Parameters values for aged (Day 5) stressed worms, as generated by the hybrid optimization algorithm. For each parameter, are indicated the mean of the values it takes in all sets of plausible solutions in which it appears, standard deviation, the difference of the mean with the parameter value for young unstressed worms (S1 Table), the % mean difference with young unstressed worms, and in how many of the plausible solutions for the specific worm population this parameter appears. With bold are the parameters which seem to be more important for the changes in Ca^2+^ transients in aged (Day 5) stressed worms compared to the reference case, either due to their change compared to the reference case, or due to their abundance in the plausible solutions.
(PPTX)
