# Supplementary figures and images for: A mathematical and computational model of the calcium dynamics in Caenorhabditis elegans ASH sensory neuron
Source: PLoS One. 2018 Jul 26;13(7):e0201302. doi: 10.1371/journal.pone.0201302 (PMC6062085; doi:10.1371/journal.pone.0201302)

## Slide 1
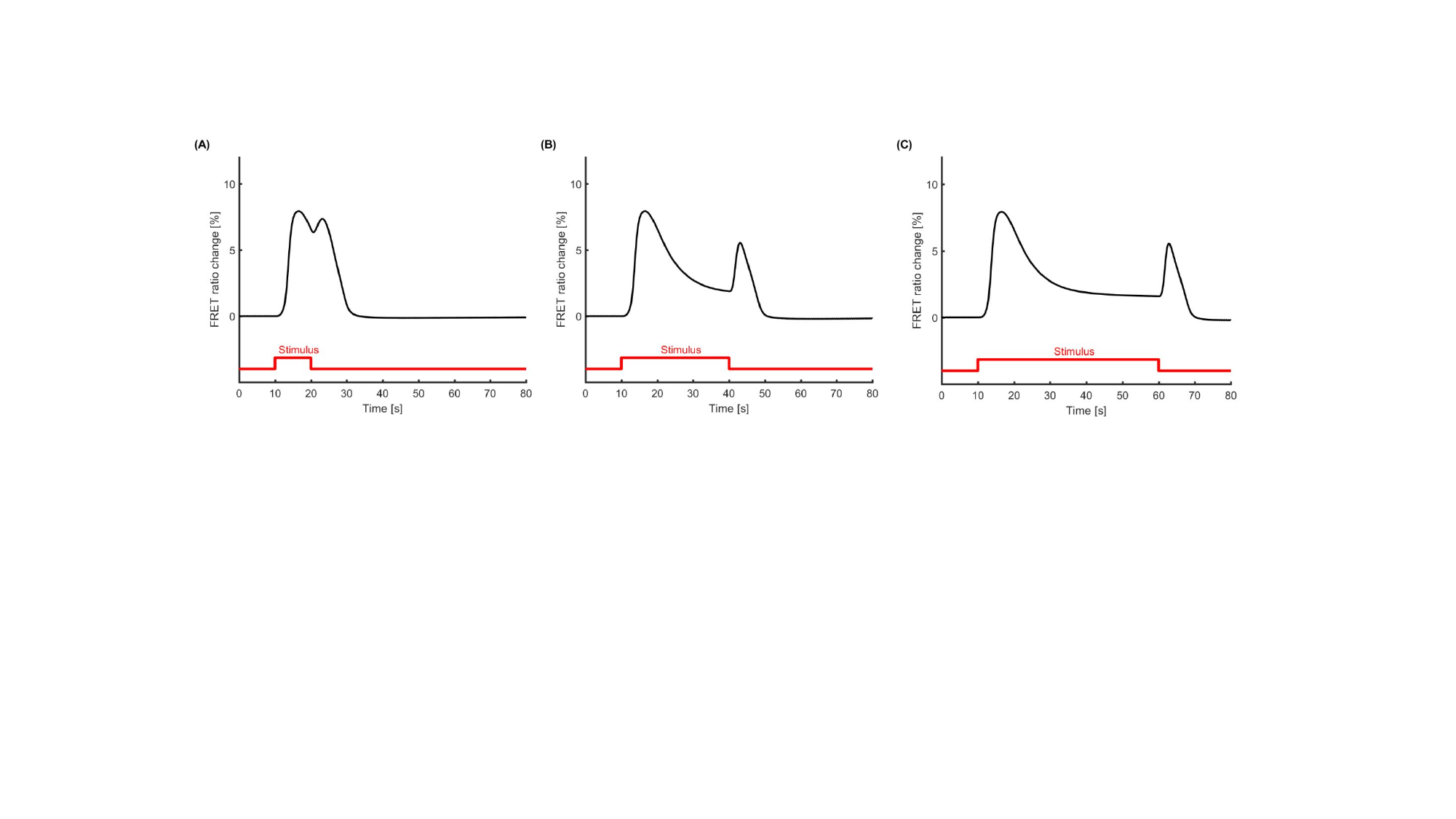

Supplement: S1 Fig — (A) A short pulse of 10sec still results in distinct peaks for “on” and “off” responses of different magnitudes, without the plateau region; (B) The Ca2+ transient induced by the pulse (30sec) delivered in the experimental data and the model results, presented here for comparison; (C) A long pulse of 50 sec results in a Ca2+ transient of similar shape with the one shown in (B). The response to the shorter stimulus in (A) includes an “off” response stronger than the one observed in (B) and (C), although still smaller than the “on” peak. All three Ca2+ transients are generated using the parameters estimated for young unstressed worms (reference case). (PPTX) [file pone.0201302.s001.pptx]

## Slide 1
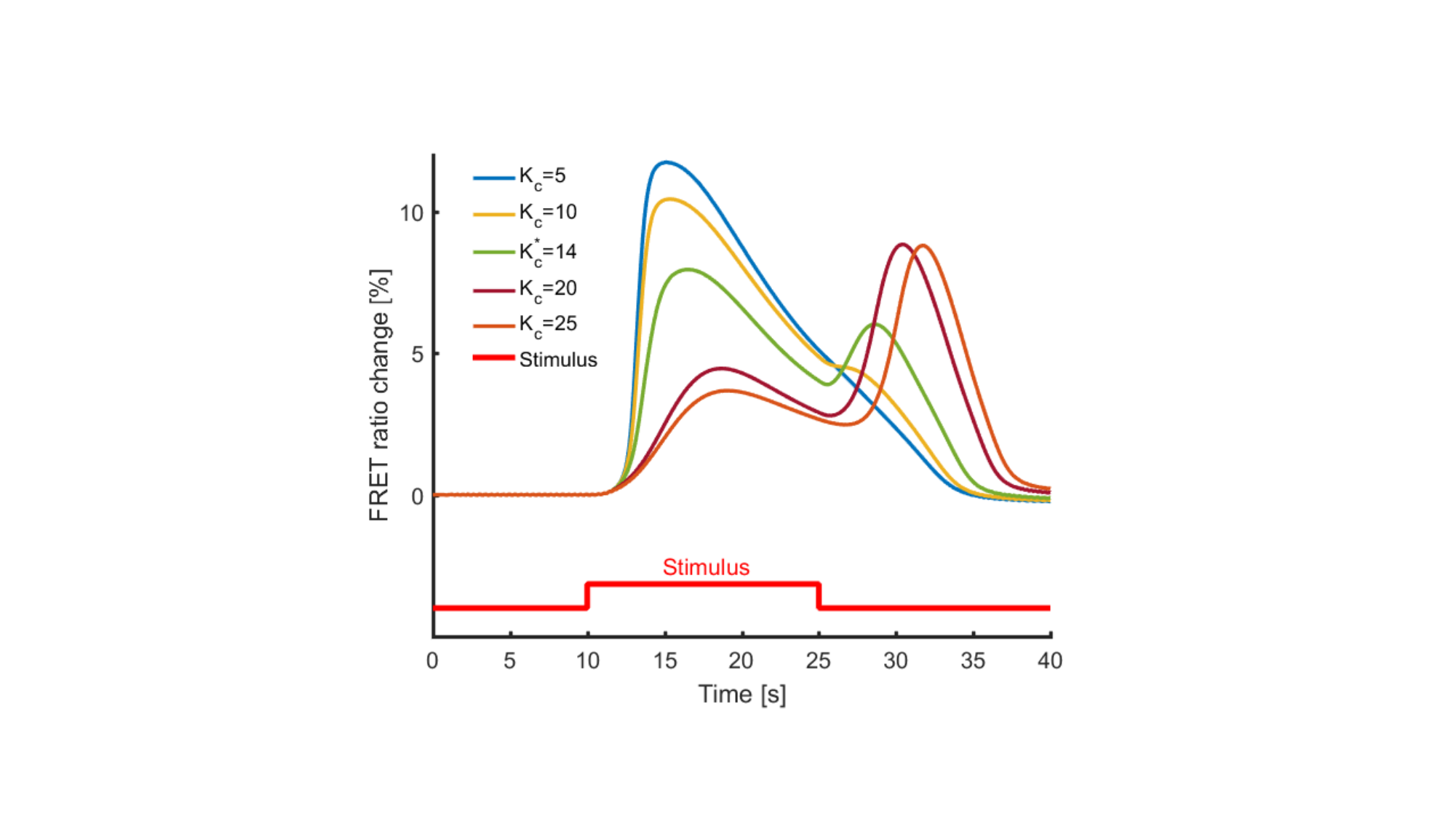

Supplement: S2 Fig — Kc in Eq 16, which affects dynamics of IP3, can be changed to control the relative magnitude of “on” and “off” response. A weaker “on” response leads to a stronger “off” response because when less Ca2+ is released from the ER during the “on” response, then there is more available to be released from ER during the “off” response. Kc* corresponds to the value of this parameter used in the model for young unstressed worms (reference case). (PPTX) [file pone.0201302.s002.pptx]

## Slide 1
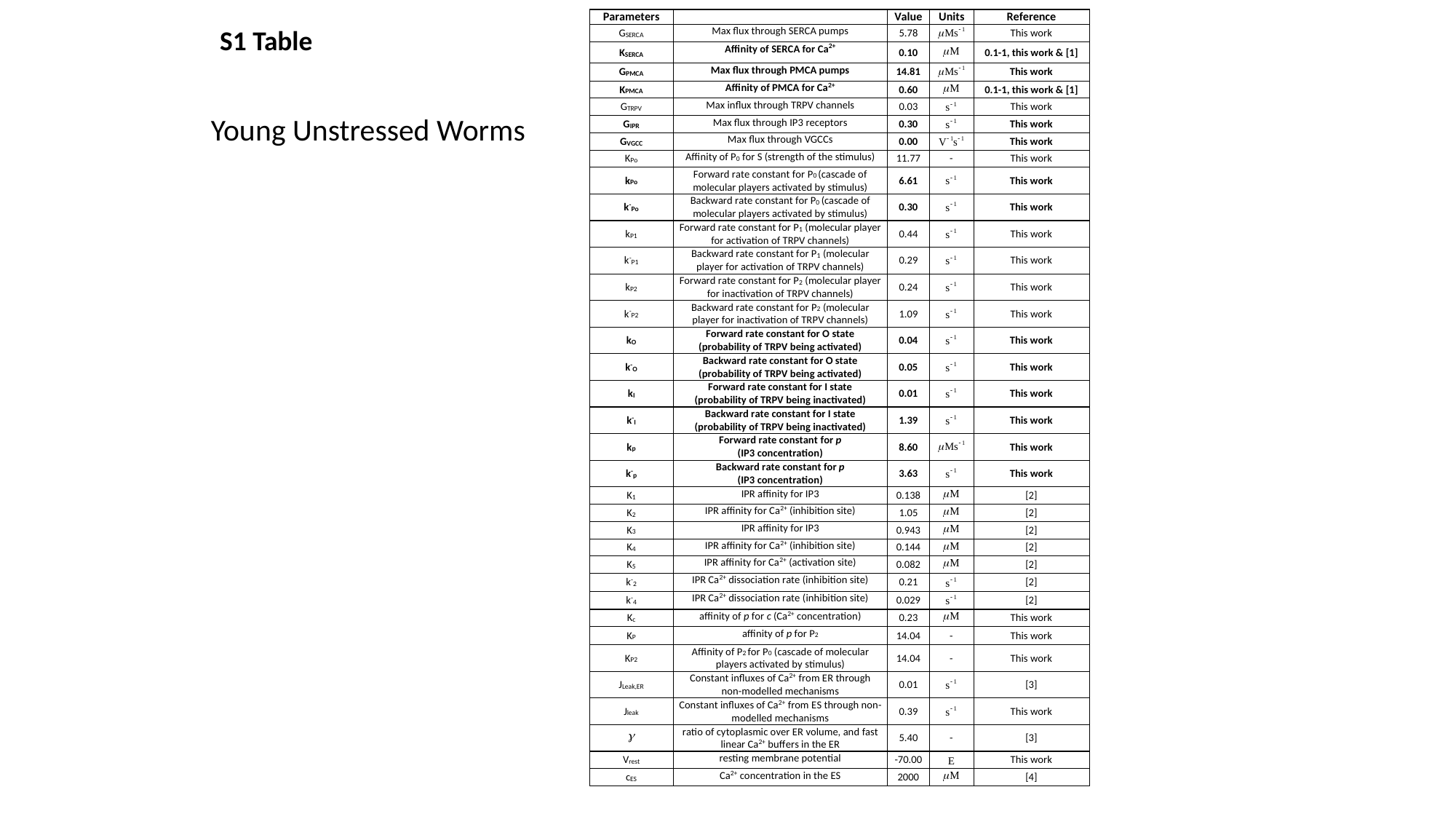

S1 Table
Young Unstressed Worms

Supplement: S1 Table — With bold are the selected parameters which are investigated for the aging and oxidative stress effect in the next three worm populations. (PPTX) [file pone.0201302.s003.pptx]
