# Supplementary material for: A mathematical and computational model of the calcium dynamics in Caenorhabditis elegans ASH sensory neuron
Source: PLoS One. 2018 Jul 26;13(7):e0201302. doi: 10.1371/journal.pone.0201302 (PMC6062085; doi:10.1371/journal.pone.0201302)
Supplement: S3 Table — For each parameter, are indicated the mean of the values it takes in all sets of plausible solutions in which it appears, standard deviation, the difference of the mean with the parameter value for young unstressed worms (S1 Table), the % mean difference with young unstressed worms, and in how many of the plausible solutions for the specific worm population this parameter appears. With bold are the parameters which seem to be more important for the changes in Ca2+ transients in aged (Day 5) unstressed worms compared to the reference case, either due to their change compared to the reference case, or due to their abundance in the plausible solutions. (PPTX) [file pone.0201302.s005.pptx]

## Slide 1
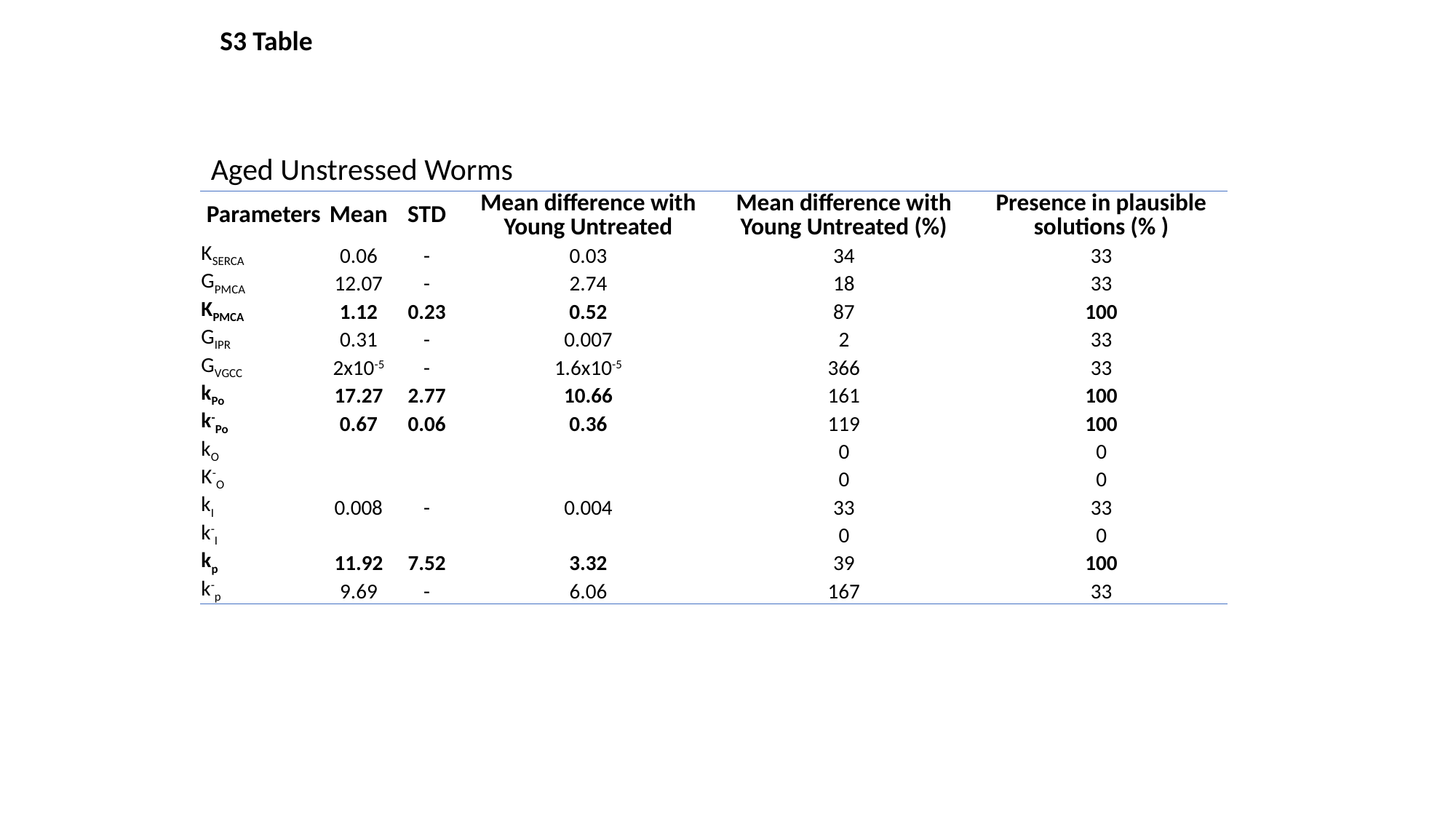

S3 Table
Aged Unstressed Worms
| Parameters | Mean | STD | Mean difference with Young Untreated | Mean difference with Young Untreated (%) | Presence in plausible solutions (% ) |
| --- | --- | --- | --- | --- | --- |
| KSERCA | 0.06 | - | 0.03 | 34 | 33 |
| GPMCA | 12.07 | - | 2.74 | 18 | 33 |
| KPMCA | 1.12 | 0.23 | 0.52 | 87 | 100 |
| GIPR | 0.31 | - | 0.007 | 2 | 33 |
| GVGCC | 2x10^-5^ | - | 1.6x10^-5^ | 366 | 33 |
| kPo | 17.27 | 2.77 | 10.66 | 161 | 100 |
| k-Po | 0.67 | 0.06 | 0.36 | 119 | 100 |
| kO | | | | 0 | 0 |
| K-O | | | | 0 | 0 |
| kI | 0.008 | - | 0.004 | 33 | 33 |
| k-I | | | | 0 | 0 |
| kp | 11.92 | 7.52 | 3.32 | 39 | 100 |
| k-p | 9.69 | - | 6.06 | 167 | 33 |
